# Study on the molecular mechanism of anti-liver cancer effect of Evodiae fructus by network pharmacology and QSAR model

**DOI:** 10.3389/fchem.2022.1060500

**Published:** 2023-01-09

**Authors:** Peng-Yu Chen, Lin-Tao Han

**Affiliations:** ^1^ Faculty of Pharmacy, Hubei University of Chinese Medicine, Wuhan, China; ^2^ Key Laboratory of Traditional Chinese Medicine Resources and Prescription, Ministry of Education, Wuhan, China

**Keywords:** Euodiae fructus, liver cancer, network pharmacology, molecular docking, QSAR model, molecular dynamics simulation

## Abstract

**Introduction:** Evodiae Fructus (EF) is the dried, near ripe fruit of *Euodia rutaecarpa* (Juss.) Benth in *Rutaceae*. Numerous studies have demonstrated its anti-liver cancer properties. However, the molecular mechanism of Evodiae fructus against liver cancer and its structure-activity connection still require clarification.

**Methods:** We utilized network pharmacology and a QSAR (2- and 3-dimensional) model to study the anti-liver cancer effect of Evodiae fructus. First, by using network pharmacology to screen the active substances and targets of Evodiae fructus, we investigated the signaling pathways involved in the anti-liver cancer actions of Evodiae fructus. The 2D-QSAR pharmacophore model was then used to predict the pIC50 values of compounds. The hiphop method was used to create an ideal 3D-QSAR pharmacophore model for the prediction of Evodiae fructus compounds. Finally, molecular docking was used to validate the rationality of the pharmacophore, and molecular dynamics was used to disclose the stability of the compounds by assessing the trajectories in 10 ns using RMSD, RMSF, Rg, and hydrogen bonding metrics.

**Results:** In total, 27 compounds were acquired from the TCMSP and TCM-ID databases, and 45 intersection targets were compiled using Venn diagrams. Network integration analysis was used in this study to identify SRC as a primary target. Key pathways were discovered by KEGG pathway analysis, including PD-L1 expression and PD-1 checkpoint pathway, EGFR tyrosine kinase inhibitor resistance, and ErbB signaling pathway. Using a 2D-QSAR pharmacophore model and the MLR approach to predict chemical activity, ten highly active compounds were found. Two hydrophobic features and one hydrogen bond acceptor feature in the 3D-QSAR pharmacophore model were validated by training set chemicals. The results of molecular docking revealed that 10 active compounds had better docking scores with SRC and were linked to residues *via* hydrogen and hydrophobic bonds. Molecular dynamics was used to show the structural stability of obacunone, beta-sitosterol, and sitosterol.

**Conclusion:**Pharmacophore 01 has high selectivity and the ability to distinguish active and inactive compounds, which is the optimal model for this study. Obacunone has the optimal binding ability with SRC. The pharmacophore model proposed in this study provides theoretical support for further screening effective anti-cancer Chinese herbal compounds and optimizing the compound structure.

## Graphical Abstract

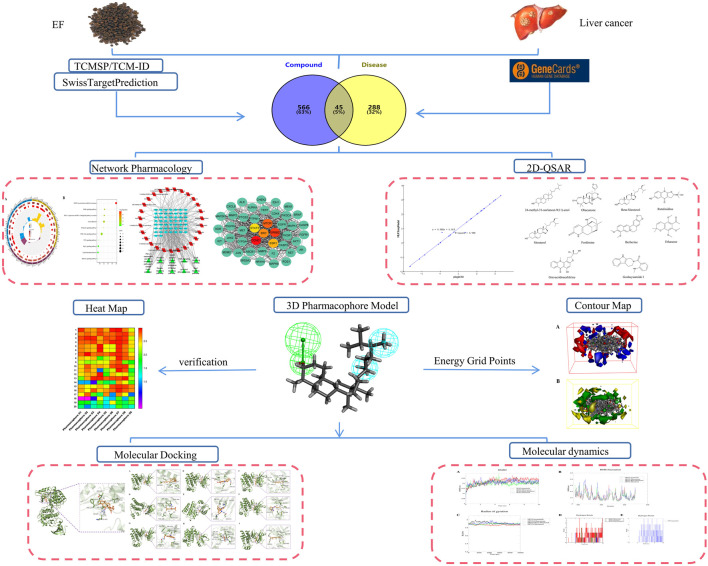

## 1 Introduction

Liver cancer, including intrahepatic cholangiocarcinoma (ICCA) and hepatocellular carcinoma (HCC), is the second-largest cause of cancer-related mortality worldwide and a significant public health issue (Kassebaum et al., 2014). Its incidence rate and mortality have increased annually, and over the past 20 years, it has become the cancer factor in the United States that has caused the biggest increase in mortality (Sia et al., 2017). In 2017, More than half of all liver cancer cases worldwide—5,70,000 new cases—occurred in China. The mortality rate was 26.26/1,00,000, and men had a greater mortality rate than women (37.55/1,00,000 vs. 14.45/1,00,000) (He et al., 2021). Cao et al. (2021) conducted a secondary analysis of cancer statistics from around the world. In 2020, cancer cases in China accounted for 24% of newly confirmed cases worldwide and 30% of cancer deaths worldwide. The death rate from liver cancer rose to second in China in 2020.

Currently, the clinical treatment of liver cancer includes surgery and drug chemotherapy. On the one hand, liver cancer has an extremely dismal prognosis; only 5%–15% of patients are candidates for surgical resection. Therefore, it is only appropriate for early-stage patients with some liver regeneration potential (Anwanwan et al., 2020). On the other hand, the most widely prescribed medication for individuals with advanced stages is the kinase inhibitor sorafenib. However, less than one-third of patients will be able to fully benefit from the course of their treatment. Sorafenib resistance is visible after use, and problems including toxicity and ineffectiveness can also result from prolonged exposure to chemotherapy medicines (El-Serag et al., 2008). Given the poor prognosis of liver cancer, scientists and physicians have been looking for new treatment options to improve patient survival.

Natural products provide distinct advantages in cancer treatment. Natural plant extracts and natural chemicals, as well as traditional Chinese medicines, have gained a lot of attention in recent years for their high-efficiency and low-toxicity anti-cancer characteristics (Cho et al., 2015). Evodiae Fructus (EF), a traditional medicinal plant, is the dried and nearly ripe fruit of the *Rutaceae Euodia rutaecarpa* (Juss.) Benth. EF contains alkaloids, terpenes, flavonoids, phenolic acids, steroids, and phenylpropanoids (Li and Wang, 2020). Modern pharmacological researches have demonstrated its biological properties, including cardioprotective, antibacterial, anti-inflammatory, and anti-tumor effects (Yoon et al., 2013; Zhao et al., 2019; Shan et al., 2020). According to several research, evodiamine, and rutaecarpine in EF had hepatoprotective properties (Yoon et al., 2013). Meanwhile, evodiamine may induce apoptosis in liver cancer cells *via* the WWOX-dependent pathway, as well as the Akt pathway and others, and then exert antitumor effects (Hu et al., 2017; Yang et al., 2017). Furthermore, EF extracts can prevent the development of a variety of cancers, including colon cancer, cervical cancer, and others (Dong et al., 2010; Park et al., 2017). However, the molecular mechanism of EF against liver cancer is currently understudied.

By combining bioinformatics and network analysis, network pharmacology is particularly well suited for the analysis of complex pharmacological mechanisms of multi-compound (Xia and Tang, 2021). The quantitative structure activity relationship (QSAR) method is widely used to investigate the relationship between the physicochemical properties of chemicals and their biological activities in order to obtain a mathematical statistical model for predicting the activity of target chemicals, with differences in structural properties leading to different bioactivities of compounds as the basic principle (Verma et al., 2010). Recently, it has been fashionable to employ QSAR pharmacophore models to investigate the structure-activity connections of TCM and natural compounds in order to uncover their biological activities (Elekofehinti et al., 2021; Li et al., 2022). It is well known that small molecule drugs frequently bind to macromolecular receptors to perform specific biological functions. Molecular docking methods have been widely used in modern drug design to investigate the conformation of ligands within the binding sites of macromolecular target proteins and to predict their binding mode and binding capacity (Ferreira et al., 2015).

Nowadays, the molecular docking method is an important technique in the field of computer-assisted drug research, and it has become an increasingly important tool for drug discovery. Molecular docking creates drugs based on receptor characteristics and the mode of interaction between receptors and drugs. It can simulate the interaction of molecules and proteins at the atomic level to elucidate the binding sites and binding characteristics of small molecules on target proteins, as well as the fundamental biochemical processes. Molecular docking can also predict ligand and receptor conformation and calculate parameters such as affinity to evaluate binding. This technique is accurate and low-cost, and it is currently used primarily for drug design and the elucidation of biochemical processes (Meng et al., 2011).

In this study, active compounds and key targets of EF were first screened using the network pharmacology method, followed by GO and KEGG analysis to investigate the molecular mechanism. The pIC50 of active compounds in EF were successfully predicted by the 2D-QSAR pharmacophore model, and the hiphop method was used to construct the 3D-QSAR pharmacophore model. Finally, molecular docking was used to confirm the binding modes of ligands to proteins. In the graphic abstract, the main concept under study is displayed.

## 2 Materials and methods

### 2.1 Network pharmacology

#### 2.1.1 Chemical compounds and targets acquisition

We use the Traditional Chinese Medicine Systems Pharmacology Database and Analysis Platform (TCMSP, http://tcmspw.com/tcmsp.php) and the Traditional Chinese Medicine Information Database (TCM-ID, http://bidd.group/TCMID/index.html) to obtain the chemical compounds in EF. The OB value (Oral bioavailability) and DL value (Drug-likeness) used as a reference for filter chemicals are 30% and .18%, respectively. Their structures were retrieved in SDF format from the PubChem database. Then we utilized the SwissTargetPrediction (http://www.swisstargetprediction.ch/) to find compound targets, entered “liver cancer” at GeneCards (https://www.genecards.org/) to find genes associated with liver cancer (score > 30), and then we analyzed the intersection targets with the aid of the venny2.1.0 platform to find the appropriate targets of EF against liver cancer.

#### 2.1.2 GO/KEGG analysis

DAVID Bioinformatics Resources (https://david.ncifcrf.gov/home.jsp) is a well-known bioinformatics resource system for functional annotation and enrichment analysis of gene lists (Sherman et al., 2022). We submitted the intersection targets of EF treatment for liver cancer to the DAVID database, chose *Homo sapiens* as the species, and obtained items from Gene Ontology (GO) and the Kyoto Encyclopedia of Genes and Genomes (KEGG). It is used to annotate biological functions and analyze signal pathways of key targets.

#### 2.1.3 C-T-P and PPI network construction

The “Compounds-Targets-Pathways” (C-T-P) network is frequently used to analyze the interactions between compounds, targets, and pathways (Chen et al., 2021). Furthermore, the analysis of Protein-protein Interaction (PPI) networks can contribute to a better understanding of disease molecular mechanisms by systematically analyzing and discovering important targets (O’Reilly et al., 2019). The PPI network was obtained using the STRING database (https://string-db.org/). The C-T-P network was built using the Cytoscape 3.9.1 software, and we ranked compounds by degree value to determine their significance. The key targets in the PPI network were examined using Cytoscape 3.9.1’s cytohubba plugin analysis.

### 2.2 Construction of the QSAR model

We obtained 45 SRC inhibitors based on the literature and the Selleck website (https://www.selleck.cn/) (Martin et al., 2006; Bain et al., 2007; Schenone et al., 2007; Hiscox and Nicholson, 2008; Fathi et al., 2019; Ma et al., 2020). The 3D structural formulas were downloaded from PubChem, and energy was minimized through batch processing using Discovery Studio Software (Discovery Studio 2019; BIOVIA; San Diego, United States). Using the “Creat Training and Test Data” function module, all SRC inhibitor compounds were randomly divided into a training set (35 compounds) and a test set (ten compounds) (Supplementary Figures S7, S8) The resulting activity values of the training and test set compounds ranged across four orders of magnitude, ensuring the model’s accuracy. Quantitative structure-activity relationships (QSAR) are a powerful computational method for analyzing data based on chemical structure. The QSAR pharmacophore model was created by establishing a statistical mathematical link between calculated chemical descriptors of molecular structure and experimentally measured values of these molecules’ biological activity, which can be used to predict biological activity with a variety of target chemical products (Muratov et al., 2020).

#### 2.2.1 Construction of the 2D-QSAR pharmacophore

In this study, 2D-QSAR models were built by calculating the molecular properties of the training and test sets using Discovery Studio software. The forward selection by partial least squares (PLS) method and the stepwise multiple linear regression (MLR) method validated by the external test set prediction method were used to build the 2D-QSAR model (Hajalsiddig et al., 2020). Furthermore, the genetic function approximation method (GFA) is an intelligent regression algorithm that simulates biological evolution and natural selection in nature. Because GFA provided a better fit to the training set, it was also commonly used for QSAR modeling (Wang et al., 2012). The pIC_50_ value is typically used to describe the biological activity of a substance, The “calculate molecular properties” module was used to calculate a number of molecular descriptors included AlogP, molecular weight, the total number of bonds, the minimum energy of conformation (kcal/mol), volume, surface area, and other properties (Dwivedi et al., 2011; Imran et al., 2015). Some statistical parameters, such as the coefficient of determination (*r* ^2^), adjusted *r* ^2^ (r^2^ adj), and prediction (PRESS) *r* ^2^ (*r* ^2^ pred), determine the accuracy of our constructed model.

#### 2.2.2 Construction of the 3D-QSAR pharmacophore

Similarly, we built 3D-QSAR models with Discovery Studio software, typically using the HipHop method. The hiphop method uses the three-dimensional structures of a series of known target inhibitors/activators to describe the common features of biological activities, develop pharmacophore models, and finally generate the best quantitative pharmacophore model for 3D querying (Wang et al., 2008). First, we screened 10 compounds with higher EF activities and imported their structures’ SDF format (3D) into Discovery Studio software. The principal value and maximumomitfeat value of each compound are then defined. Compounds were designated as active when their IC_50_ was less than 1μM, the Principal value was set to 2, and the Maxomitfeat value was set to 0. When their IC_50_ was greater than 1 μM, both the Principal and Maxomitfeat values were set to 1 (Rampogu et al., 2018). Feature mapping is used to identify compound feature elements and investigate molecules that contain those main feature elements. Furthermore, we performed pharmacophore feature element selection to select those included in the HypoGen module, which generally included five types of feature elements: hydrophobic, hydrogen bond donor (Donor), hydrogen bond acceptor (Acceptor), positively charged ion center (Ionizable Positive), and aromatic ring center (Ring Aromatic). Next, the Maximum Conformation was set to 255 and the Energy Threshold to 10 (Jiang et al., 2016). Finally, the compounds from the test set were used to validate the pharmacophore model.

The 3D structure of a set of ligands was used to calculate the potential energy in the discovery Studio software’s 3D-QSAR method, and the potential energy was then used as a descriptor to build the model. Such a model correlates the molecular field and activity and links the three-dimensional structure and biological activity (Ahmed et al., 2017). The equation is as follows:

$$Activity\left(Predicted\right)=\sum_{i=1}^{NEP}CEP\left(i\right)VEP\left(i\right)+\sum_{i=1}^{NVDW}CVDW\left(i\right)VVDW\left(i\right)$$

where, N_EP_ represents the number of descriptors of electrostatic potential (EP), CEP(i) is the model coefficient of EP descriptor, and VEP(i) is the electrostatic potential value on the grid points. Furthermore, N_VDW_ is the number of descriptors of Van Der Waals (VDW) interactions, C_VDW_(i) is the model coefficient of VDW descriptor I, and V_VDW_ is the VDW interaction potential energy on the grid points. In summary, the predicted activity of a compound is the linear sum of the model coefficients multiplied by their corresponding grid values.

### 2.3 Molecular docking

Molecular docking is currently a key method in drug design since it enables research of ligand binding modalities, stable ligand receptor complex intermolecular interactions, and binding energy predictions (Huang and Zou, 2010). The top ten active compounds in the pharmacophore model were chosen as ligand molecules, and the key target SRC was chosen as the target protein for AutoDock molecular docking tests. We obtained SRC crystal structure 1BYG from the PDB database. To improve reliability, we chose reference drugs such as Ponatinib and Dasatinib, which are SRC inhibitors that are already in clinical use (Ceppi et al., 2012; Roskoski, 2015). Furthermore, STAUROSPORINE (STU), the original SRC ligand, is used as a control (1BYG).

The SRC protein structure (1BYG) was obtained from the Protein Data Bank (https://www.rcsb.org/). First, the proteins were processed with PyMOL software, which removed water molecules and extracted ligands while recording the docking site coordinates (Getbox plugin) of the chosen ligands. The small molecules were then preprocessed with AutoDockTools1.5.6, and the proteins were hydrogenated and charged. After that, one profile (including protein and ligand structure files as well as docking site information) was created for AutoDock Vina docking. Finally, information about the docking results was obtained from the PLIP website (https://plip-tool.biotec.tu-dresden.de/plip-web/plip/index), and the docking results were visualized using PyMOL software.

### 2.4 Molecular dynamics simulation

GROMACS is used for molecular dynamics (MD) simulations of protein-ligand complexes. This experiment included compounds with high docking scores that formed complexes with proteins such as 1BYG-Obacunone, 1BYG-Beta-sitosterol, and 1BYG-Sitosterol. Meanwhile, 1BYG-Ponatinib was used as a control. We used the Acpype portal and the AMBER force field to generate topology files for the ligands, with SPC as the water model. The relevant scripts were run to add water models, ions, and balance charges. The systems were stabilized by running energy minimization (100 ps) with the steepest descent algorithm. After that, we used the Verlet algorithm for NVT equilibration for 100 ps (2fs steps) and the Berenson algorithm for NPT equilibration for 100 ps (2fs steps) (Adelusi et al., 2021). To ensure that atoms did not jump out of the PBC, the “trjconv” module was used for system processing (periodic boundary conditions). The stability was assessed using root mean square deviation (RMSD), root mean square fluctuation (RMSF), radius of gyration (Rg), and hydrogen bond analysis.

## 3 Results and discussion

### 3.1 Network pharmacology analysis

#### 3.1.1 Chemical compounds and targets acquisition

With OB 30% and DL .18 as thresholds, 27 compounds from EF (Supplementary Table S1) were screened from the TCMSP and TCM-ID databases. SwissTargetPrediction identified a total of 611 compound targets. Genecards identified 333 liver cancer targets, and Venn analysis revealed 45 intersecting targets that could be used by EF to play pharmacological roles in the anti-liver cancer process (Supplementary Figure S1; Supplementary Table S2).

#### 3.1.2 GO/KEGG analysis results

**FIGURE 1:**
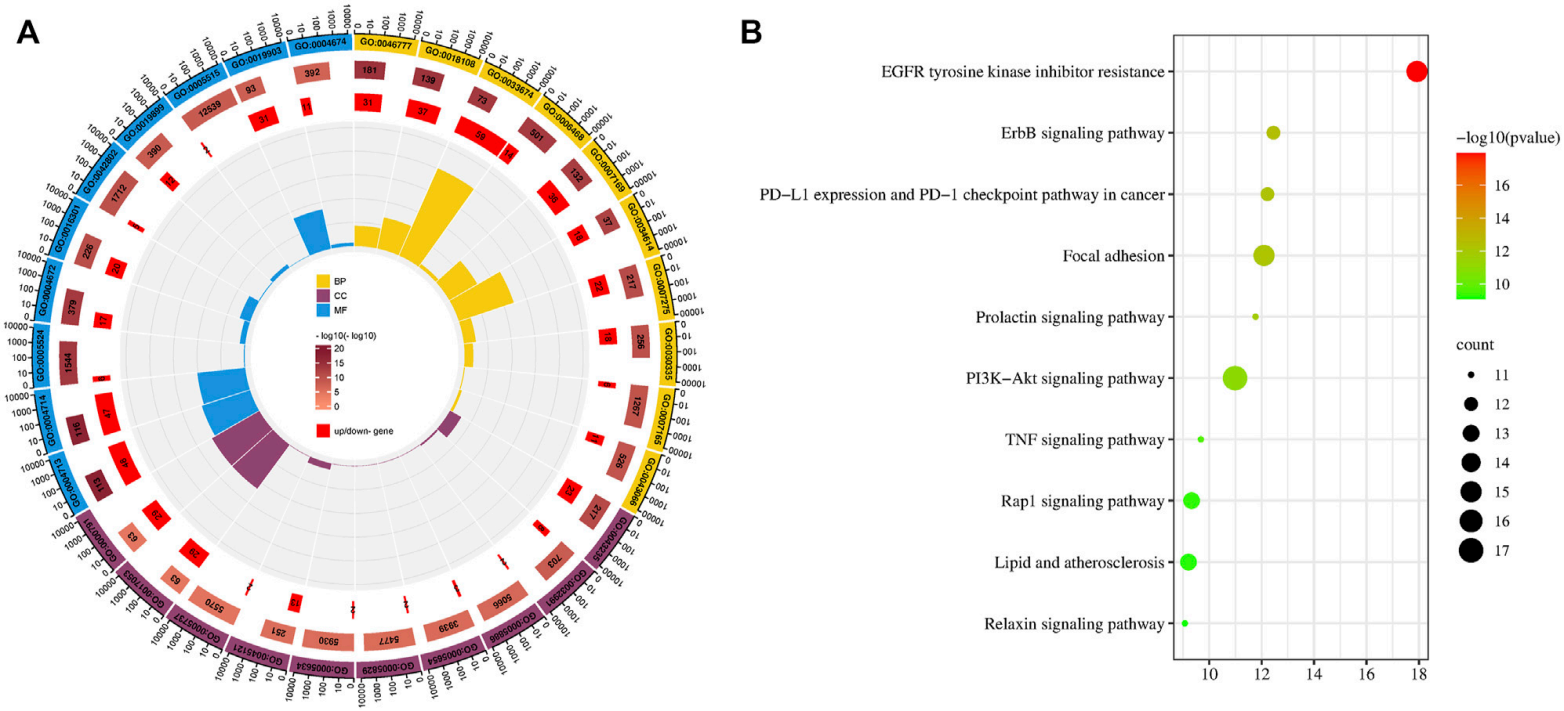
GO and KEGG analysis. **(A)** Top ten item of GO enrichment analysis of biological processes (BP), cell components (CC), and molecular functions (MF). **(B)** Bubble diagram of KEGG analysis.

We screened the top ten items in the GO and KEGG enrichment analyses in ascending order of *p*-value (Figure 1). The results of the Go analysis indicate that the biological processes (BP) mainly involve the development of multicellular organisms, ellular response to reactive oxygen species, protein autophosphorylation, peptidyl-tyrosine phosphorylation, positive regulation of kinase activity, protein phosphorylation, and transmembrane receptor protein tyrosine kinase signaling pathway. Cell Components (CC) include the receptor complex, the macromolecular complex, the plasma membrane, the nucleoplasm, the cytosol, the nucleus, the membrane raft, the cytoplasm, and so on. Protein tyrosine kinase activity, transmembrane receptor protein tyrosine kinase activity, ATP binding, protein kinase activity, kinase activity, identical protein binding, enzyme binding, protein binding, and so on are examples of Molecular Functions (MF) (Supplementary Table S3).

A total of 138 pathways with significant meaning (P0.05) were enriched by KEGG analysis. The top ten key pathways include EGFR tyrosine kinase inhibitor resistance, ErbB signaling pathway, PD-L1 expression and PD-1 checkpoint pathway in cancer, Focal adhesion, Prolactin signaling pathway, PI3K-Akt signaling pathway, TNF signaling pathway, Rap1 signaling pathway, Lipid and atherosclerosis, and others. The EGFR and ErbB signaling pathways are the highest ranked in KEGG and are upstream in other pathways. EGFR and ErbB are members of a family of cell membrane protein receptors that can receive stimuli and send signals downstream, triggering a series of regulatory processes for both life activities and diseases. EGFR inhibition has been shown in studies to inhibit HCC cell survival, migration, and invasion (Chen et al., 2019; Jin et al., 2021). The ErBB-PI3K-AKT pathway can promote the growth and spread of hepatocellular carcinoma (Ni et al., 2020). Overexpression of focal adhesion kinase (FAK) occurs frequently in human HCC tissues, and simultaneous overexpression of FAK increases AR expression, which leads to HCC formation in mice (Shang et al., 2019). Certainly, there is also evidence that the Prolactin signaling pathway, the PI3K-Akt signaling pathway, the TNF signaling pathway, and other pathways play important roles in the process of liver cancer (Dumaual et al., 2012; Meng et al., 2021; Miethe et al., 2021).

#### 3.1.3 Network analysis

**FIGURE 2:**
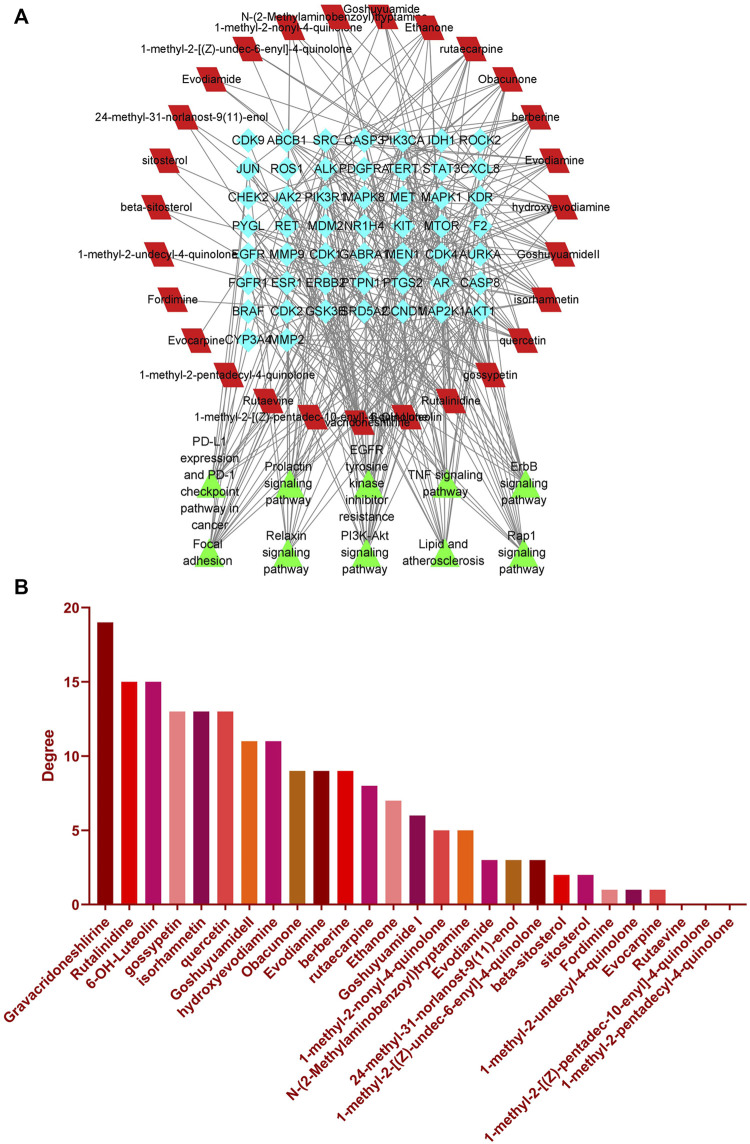
Network construction. **(A)** C-T-P network. **(B)** Degree value distribution histogram of EF compounds in C-T-P network.

We can further investigate the relationship between EF compounds, targets, and pathways by building the C-T-P network with Cytoscape 3.9.1. The C-T-P network, as shown in Figure 2A, demonstrates interactions between compounds-targets-pathways. This network contains 314 edges that represent interactions between nodes. The network also included 88 nodes: 27 shared by red square nodes representing compounds, 51 shared by blue diamond nodes representing targets, and 10 shared by green triangle nodes representing signaling pathways. The higher the degree value of the node, the more critical this node is. This network reflects the characteristics of multi-compound and multi-target interactions in TCM. Furthermore, it demonstrated that these chemicals have a better potential for bioactivity against liver cancer. In the C-T-P network, higher degree values for compounds such as gravacridoneshlirine, gossypetin, quercetin, isorhamnetin, obacunone, and 6-OH-luteolin. It suggest a greater likelihood that they may have potential liver cancer-preventing actions (Figure 2B). Meanwhile, we screened critical targets based on their degree values (Supplementary Table S4). The results showed that SRC, which was thought to be the crucial target in this investigation, had the greatest degree value.

**FIGURE 3:**
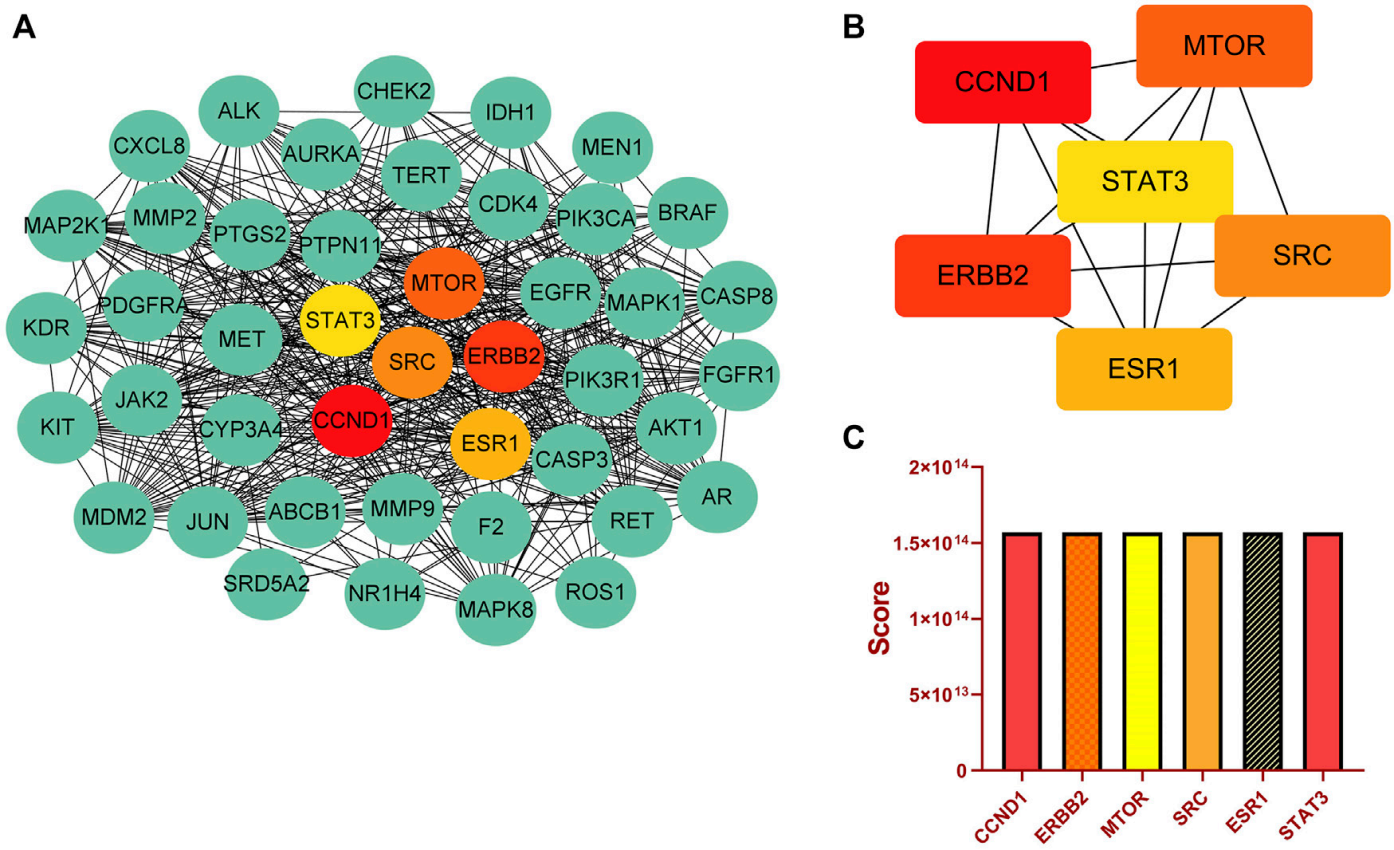
Key targets analysis. **(A)** PPI network. **(B)** Top six key targets derived from cytoHubba’s MCC algorithm. **(C)** Scores for the top six key targets.

Moreover, we identified important target proteins in the PPI network using the Cytoscape cytohubba plugin. There are eleven computational methods available to Cytohubba, which can examine the key nodes in bioinformatic networks. At the moment, MCC is regarded as being the best method (Chin et al., 2014). In addition, The PPI network showed high degree values for CCND1, ERBB2, MTOR, SRC, ESR1, and STAT3 (Figures 3A, B). Histograms depict the score values of these targets (Figure 3C; Supplementary Table S5). We discovered that SRC not only plays an important role in the C-T-P network, but also ranks high in the PPI network. SRC has a high score in comparison to the other core proteins in the PPI network, and there are close interactions. Using KEGG analysis, we discovered that SRC is involved in a number of significant signaling pathways. EGFR/ErbB-2 can activate downstream SRC proteins, and SRC can then encourage liver cancer cell growth and metastasis through focal adhesion and the PI3K-Akt signaling pathway (Ren and Schaefer, 2002; You et al., 2018; Mo et al., 2020; Luo et al., 2021). Meanwhile, a large body of literature has reported on the role of SRC in liver cancer and has shown that SRC could be a therapeutic target in liver cancer (Tong et al., 2015; Zhao et al., 2020; Yang et al., 2021). Additionally, Liao et al. (2014) demonstrated through experiments that EF extract might reduce SRC expression to reduce hepatotoxicity. Therefore, we believe SRC was the most important key target in this study.

### 3.2 2D-QSAR model analysis

#### 3.2.1 Construction of 2D-QSAR pharmacophore model

To build the 2D-QASR model, we used the GFA, MLR, and PLS linear fitting algorithms in the Discovery Studio software. The linear regression equations (Supplementary Figures S3–S5) revealed that the correlation regression coefficients *r* ^2^ of the three algorithms (GFA, MLR, and PLS) were .912, .988, and .800, respectively, demonstrating their good predictive ability and prediction accuracy for pharmacophore models, particularly MLR. Then, using these three techniques, we created 2D-QSAR models to forecast the biological activities (pIC_50_) of the compounds in the test set. The results showed that (Table 1) the predicted pIC_50_ values of the compounds based on GFA and PLS algorithms showed relatively large deviations from the experimental pIC_50_ values, indicating lower accuracy.

**TABLE 1:** Based on the 2D-QSAR test set compound experimental and predicted activity pIC50 (μM).

Training no.	Experiment pIC_50_	Predicted GFA	Residual^a^	Predicted MLR	Residual^b^	Predicted PLS	Residual^c^
1	1.046	−.676	1.722	.603	.443	3.213	2.167
2	2.699	2.858	.159	1.958	.741	2.661	.038
3	−1.758	−1.285	.474	−1.329	.429	−.898	.861
4	2.301	1.271	1.030	2.049	.252	2.399	.098
5	1.695	.968	.727	1.498	.197	1.082	.613
6	3.000	2.858	.142	3.255	.255	2.187	.813
7	−.146	.662	.808	−.427	.281	.488	.634
8	2.046	1.783	.263	2.543	.497	2.148	.102
9	3.301	4.176	.875	3.365	.064	2.226	1.075
10	.208	.968	.760	.695	.488	.899	.691
*r* ^2^		.9117		.988		.800	
*r* ^2^ (adj)		.8928		.981		.787	
*r* ^2^ (pred)		.8564		.975			

^a^ GFA-Experiment pIC50.

^b^ MLR-Experiment pIC50.

^c^ PLS-Experiment pIC50.

#### 3.2.2 Prediction of EF activity from 2D-QSAR models

**FIGURE 4:**
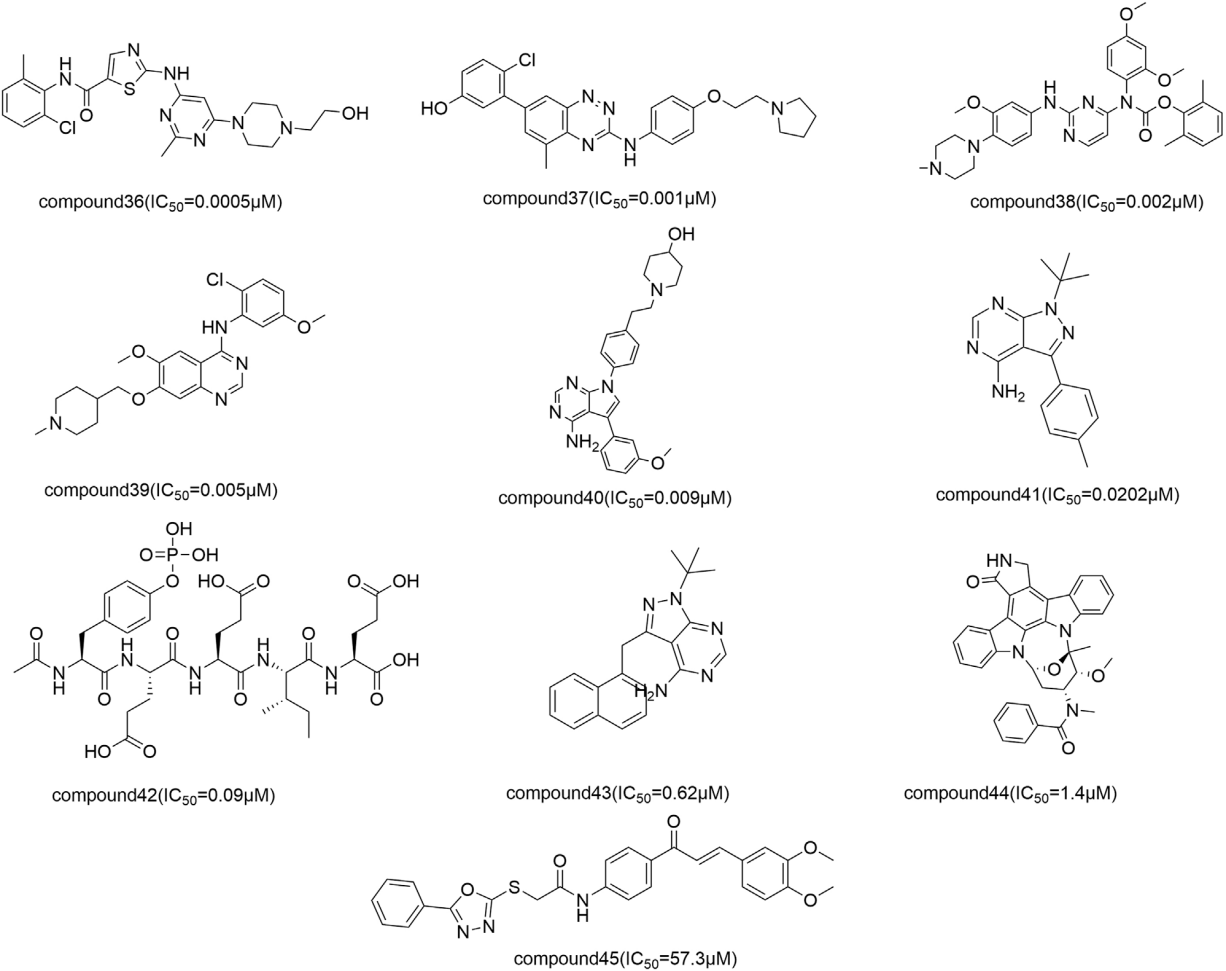
Structural of top 10 highly active compounds in EF.

The activity of the EF compounds screened was predicted by our 2D-QSAR model using the MLR method (Table 2). The top highly active compounds were 24-methyl-31-norlanost-9(11)-enol, Obacunone, Beta-Sitosterol, Berberine, Sitosterol, Rutalinidine, Fordimine, Ethanone, Gravacridoneshlirine, and Goshuyuamide I, with pIC_50_ values of 1.733, 1.491, 1.374, 1.374, .725, .470, .318 (Figure 4). Several studies have confirmed the therapeutic effects of some of these compounds in the treatment of liver cancer. Obacunone in mandarin (*Citrus reticulata* Blanco) has been shown in studies to inhibit a variety of human cancer cell lines, including leukemia (HL-60), ovarian cancer (SKOV-3), cervical cancer (Hela), gastric cancer (NCI-SNU-1), liver cancer (HepG2), and breast cancer (MCF-7). Obacunone had an IC_50_ of 65.13 ± 5.39 μm against HepG2 cells in an MTT assay (Tian et al., 2001). Mary (Ditty and Ezhilarasan, 2021) found that *β*-Sitosterol can increase cellular ROS levels, causing cell membrane damage and mitochondrial toxicity, as well as promoting HepG2 cell apoptosis. Berberine is an alkaloid that has been shown to inhibit the growth of various cancers. Ren et al. (2022) discovered that berberine inhibited hepatocarcinogenesis in mice by antagonizing the ATX-LPA-LPAR2-P38-leptin axis. Liu and Bai (2020) discovered that Sitosterol has effect on liver cancer with the help of analysis of network pharmacology. Ultimately, we screened out the top 10 compounds based on pIC_50_, which may have better anti-liver cancer activity.

**TABLE 2:** The activity pIC50 (μM) of PD Based on the 2D-QSAR model.

Pubchem CID	Compound	Predicted (MLR) pIC_50_
5319810	1-methyl-2-[(Z)-undec-6-enyl]-4-quinolone	−1.059
13967189	1-methyl-2-nonyl-4-quinolone	−.674
5319811	1-methyl-2-undecyl-4-quinolone	−.880
162926950	24-methyl-31-norlanost-9(11)-enol	1.733
5281642	6-OH-Luteolin	−1.040
12457	Berberine	.318
222284	beta-Sitosterol	1.374
624052	Ethanone	.318
5317303	Evocarpine	−1.266
189454	Evodiamide	.023
442088	Evodiamine	−.056
58757248	Fordimine	.725
5317827	Goshuyuamide_I	.142
5317828	GoshuyuamideII	−.265
5280647	gossypetin	−1.035
163102888	Gravacridoneshlirine	.151
56967381	hydroxyevodiamine	.005
5281654	Isorhamnetin	−.944
5319506	N-(2-Methylaminobenzoyl)tryptamine	−.393
119041	Obacunone	1.491

### 3.3 3D-QSAR model analysis

#### 3.3.1 Construction of the 3D-QSAR model by the HipHop method

**FIGURE 5:**
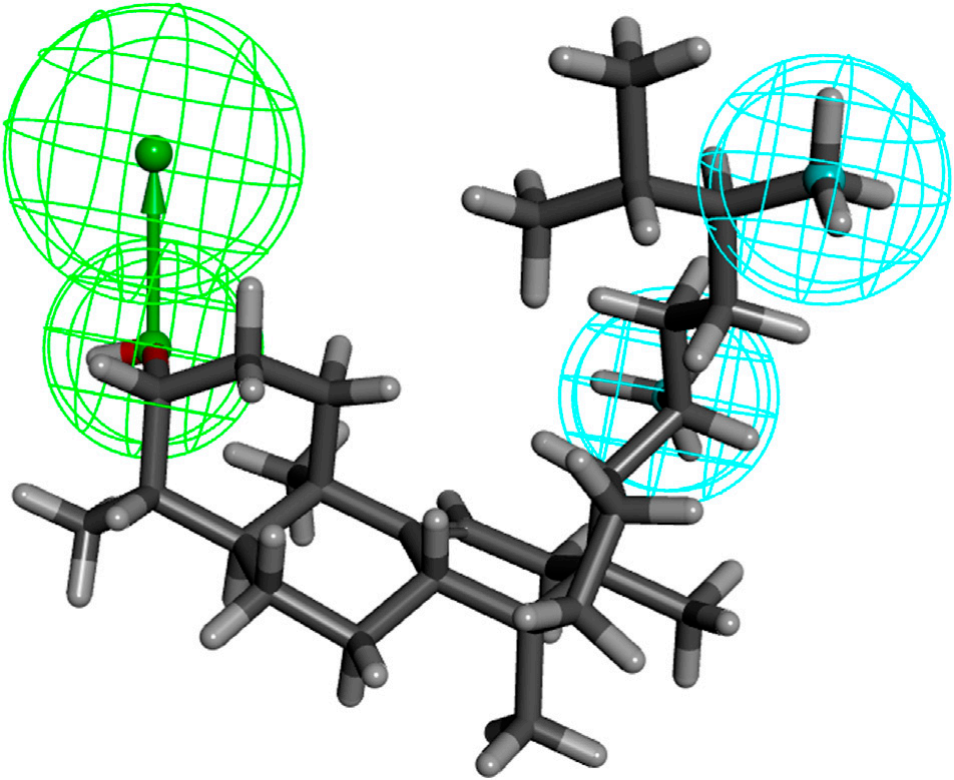
The three-dimensional structure of “24-methyl-31-norlanost-9(11)-enol” was used as an example to display “pharmacophore 01” (blue indicates hydrophobic features, green indicates hydrogen bond accepter features).

We investigated the common chemical properties of the compounds and built a 3D-QSAR model by superimposing the three-dimensional molecular structure. The HipHop method of the Discovery Studio software was used to generate nine 3D-QSAR pharmacophore models. Table 3 displays the pharmacophore model’s matching parameters with the ten active compounds. According to the results, the 01 pharmacophore model had the highest rank score value of 57.220. The “HHA” shown in its “Feature” item indicates that it has two hydrophobic features and one hydrogen bond acceptor feature. The pharmacophore features in this model were directly matched to ten small molecules, and the Partial Hit revealed no partial matches to the pharmacophore for the ten small molecules. Furthermore, Max Hit indicated that three pharmacodynamic features could all be matched. The spatial distribution of Pharmacophore 01 is depicted in Figure 5. (Supplementary Figure S6 showed the spatial distribution of pharmacophore 02–09). As a result, we chose Pharmacophore 01 for further analysis and validation as the best constructed in this study.

**TABLE 3:** Common characteristic parameters of active compounds displayed by pharmacophore models.

Pharmacophore	Feature	Rank	Direct hit	Partial hit	Max hit
01	HHA	57.220	1111111111	0000000000	3
02	HHA	57.042	1111111111	0000000000	3
03	HHA	55.151	1111111111	0000000000	3
04	HHA	52.597	1111111111	0000000000	3
05	HHA	49.170	1111111111	0000000000	3
06	HHA	48.528	1111111111	0000000000	3
07	HHA	42.982	1111111111	0000000000	3
08	HHA	41.428	1111111111	0000000000	3
09	HHA	32.624	1111111111	0000000000	3

#### 3.3.2 Construction of the 3D-QSAR model based on steric and electrostatic fields of small molecules

**FIGURE 6:**
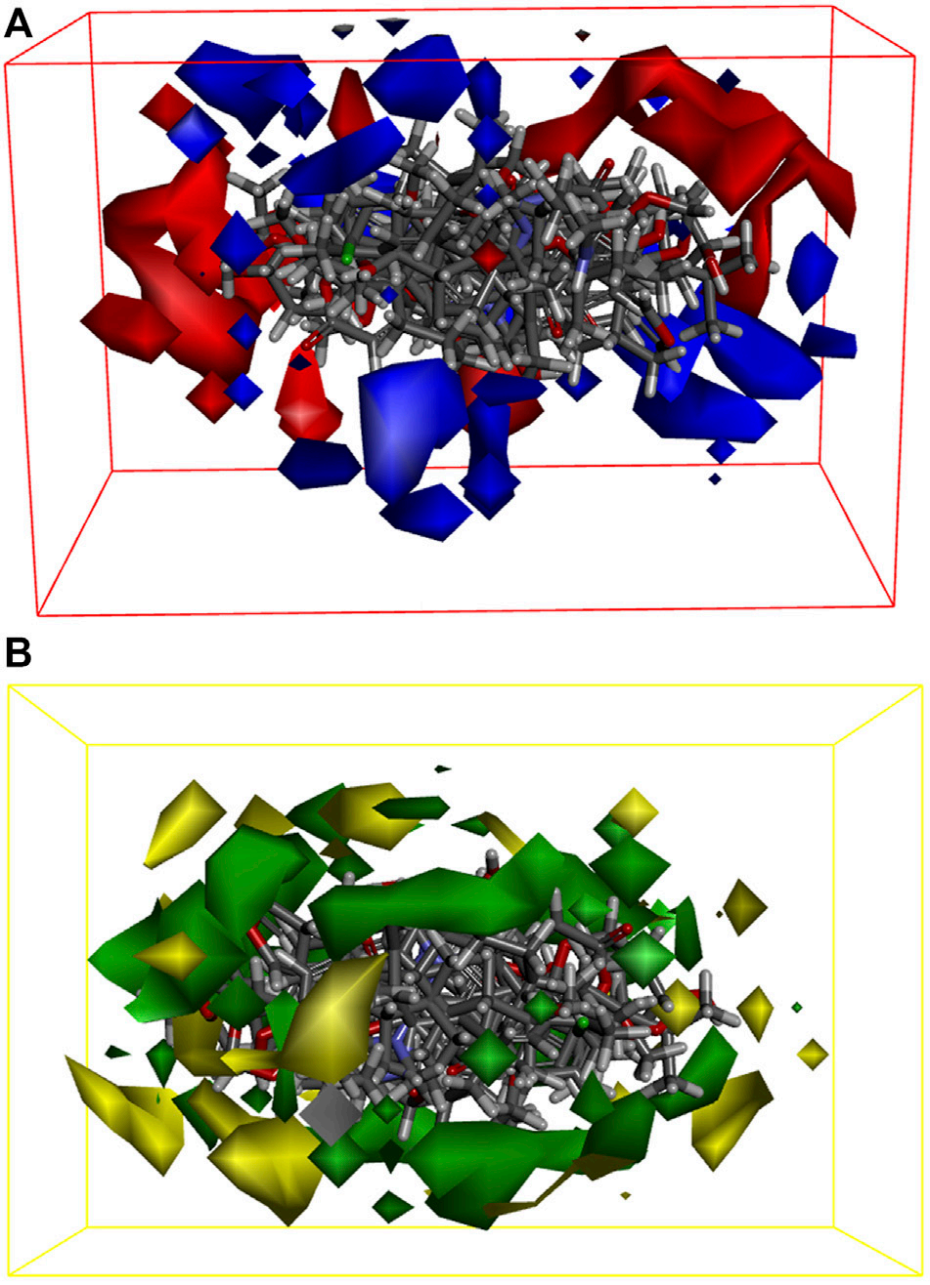
3D-QSAR models with energy grid points as descriptors. **(A)** Small molecule matching to electrostatic field coefficient isoelectric maps in the model; **(B)** Small molecule matching to stereo field coefficients isoplot in model.

We built 3D-QSAR models based on the steric and electrostatic fields of the small molecules to investigate the non-covalent interactions in the structure of EF active compounds. Our 3D-QSAR model incorporates and visualizes the common important structural characteristics of a series of active compounds. The contour plots of the electrostatic field coefficients of the small molecules that match the 3D-QSAR models are shown in Figure 6A. In this system, the more negatively charged the substituents in the red region and the more positively charged the substituents in the blue region, the higher the activity of the compound. The contour plots of the steric field coefficients of small molecules that match the 3D-QSAR model are shown in Figure 6B. In this system, the yellow region indicates that increasing the volume of substituents is detrimental to compound activity, whereas the blue region indicates that increasing the volume of substituents is beneficial to compound activity. The results showed that the electrostatic effects of the substituents, as well as the spatial distribution of the functional groups of the active compounds, affected their biological activities and may have contributed to the binding of key target proteins for liver cancer. Based on the force field information, this part of the study can help us screen and optimize the active compounds in EF.

#### 3.3.3 Verification of 3D-QSAR pharmacophore model

**FIGURE 7:**
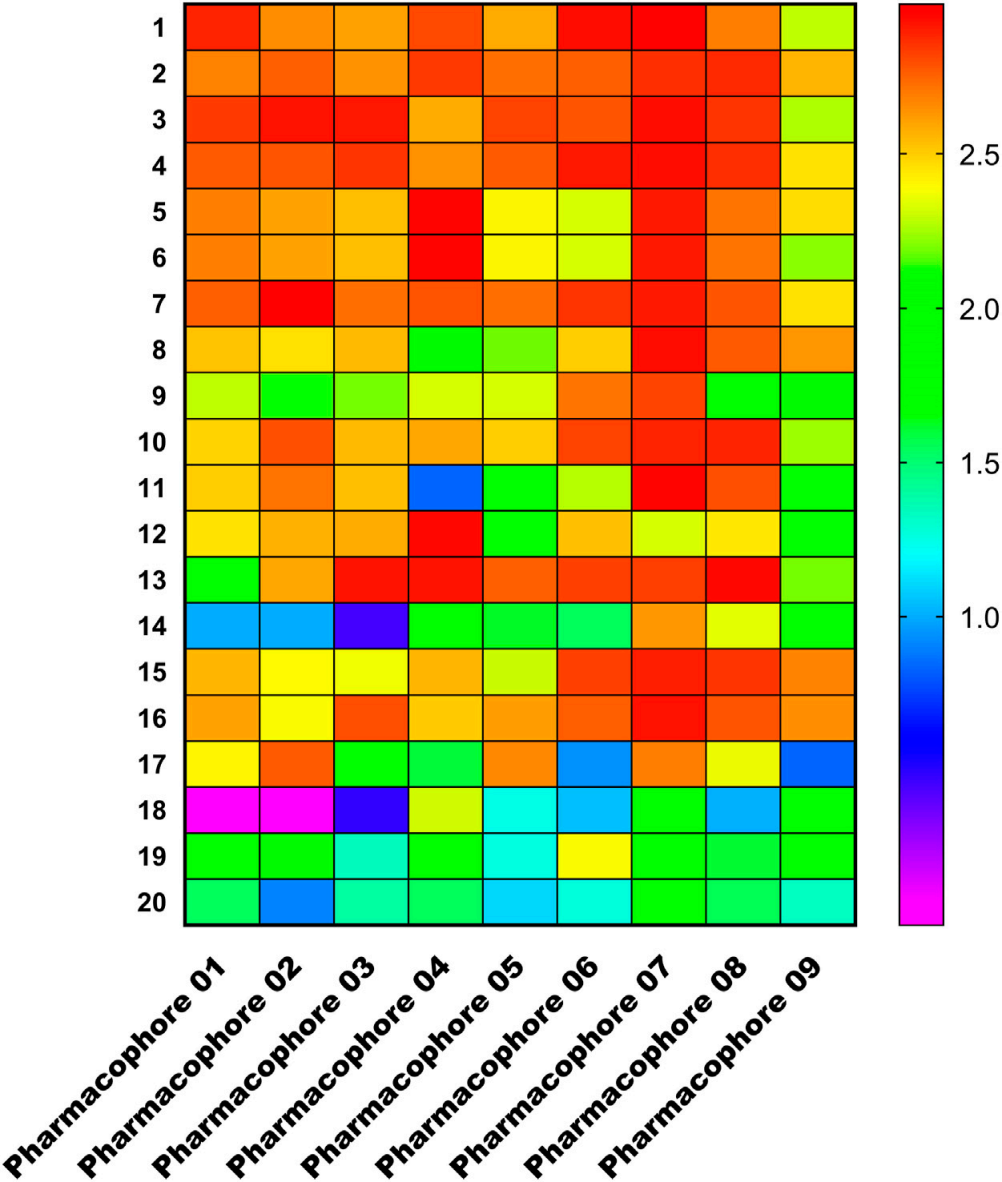
Heat map of the training set compounds predicted by the nine pharmacophore models.

We chose the top 20 training set compounds based on activity to verify that pharmacophores can correctly distinguish between active and inactive molecules. Figure 7 depicts a matching heatmap of the pharmacophore with the training set compounds, where the ordinate represents the 20 training ensemble scores ranked in order of activity, the abscissa represents the pharmacophore 01–09, and the Fitvalue ranges from low to high, as indicated by the gradual purple to red color change. The darker the red color of each rectangular block in the figure, the greater the Fitvalue, thus the greater the corresponding compound activity. In theory, the active compounds in the training set should be red and orange, while the inactive compounds should be blue and purple, indicating that this pharmacophore can distinguish between active and inactive compounds. We discovered that the Fitvalue of the more active compound in pharmacophore 01 was higher than that of the less active compound, and that its fitvalue had a certain trend, indicating that pharmacophore 01 had ability to discriminate between active and inactive compounds. Pharmacophore 01 had the highest rank value.

Additionally, we used receiver operating characteristic (ROC) curve analysis to validate the pharmacophore (Al-Sha’er et al., 2022; Taha et al., 2014). ROC plots are a quantitative measure of whether a test can distinguish between two populations (typically active/inactive compounds). It can be compared to other data sets. The area under the curve was used to evaluate test accuracy in ROC analysis (AUC). A test set of active and inactive compounds was used in our 3D-QSAR model to validate the pharmacophore model’s selectivity. On the one hand, sensitivity is defined as the model’s ability to identify positives, namely the extent to which an active molecule is distinguished from the inactive, or the proportion of the predicted active that is actually active:

$$Sensitivity=TP\;/\;\left(TP+FN\right)$$

Specificity, on the other hand, is the ability of the model to determine negatives, or its discriminatory power for inactive compounds:

$$Specific\;TN\;/\;\left(TN+FP\right)$$

ROC analysis results show that (Supplementary Figure S9; Supplementary Table S6), the ROC score of the pharmacophore 01 model is .768, and the sensitivity and specificity scores are both high. We believe pharmacophore 01 is the most effective pharmacophore hypothesis for distinguishing between active and inactive compounds. Therefore, we considered pharmacophore 01 was the best 3D-QSAR pharmacophore model. This means that pharmacophore model 01 may have anti-cancer effects on EF. The Fitvalue of the other pharmacophore models is shown in Table 4.

**TABLE 4:** Matching results of nine pharmacophore models for compounds of the training set.

Compound no.	01	02	03	04	05	06	07	08	09
1	2.886	2.649	2.604	2.805	2.585	2.934	2.965	2.682	2.289
2	2.677	2.754	2.638	2.838	2.718	2.757	2.858	2.874	2.559
3	2.834	2.924	2.912	2.583	2.818	2.777	2.931	2.851	2.261
4	2.767	2.782	2.846	2.640	2.764	2.911	2.933	2.866	2.450
5	2.681	2.598	2.535	2.956	2.399	2.325	2.905	2.708	2.462
6	2.681	2.598	2.535	2.956	2.399	2.325	2.905	2.708	2.219
7	2.756	2.983	2.717	2.787	2.719	2.850	2.903	2.773	2.453
8	2.526	2.457	2.543	1.880	2.183	2.498	2.933	2.767	2.632
9	2.284	2.126	2.194	2.321	2.326	2.706	2.810	2.037	1.817
10	2.482	2.788	2.544	2.594	2.499	2.818	2.889	2.881	2.242
11	2.502	2.711	2.530	.836	2.035	2.271	2.949	2.790	1.660
12	2.444	2.568	2.582	2.942	1.874	2.529	2.328	2.435	1.996
13	2.034	2.593	2.919	2.919	2.755	2.824	2.827	2.938	2.192
14	1.000	1.000	.455	1.978	1.629	1.539	2.625	2.345	1.933
15	2.552	2.391	2.364	2.557	2.303	2.822	2.899	2.845	2.671
16	2.601	2.387	2.797	2.504	2.610	2.761	2.923	2.781	2.644
17	2.404	2.766	1.959	1.600	2.662	.935	2.684	2.358	.839
18	.000	.000	.485	2.307	1.239	1.046	1.929	1.008	2.048
19	1.806	1.810	1.338	1.971	1.254	2.380	1.868	1.605	2.003
20	1.533	.903	1.395	1.541	1.100	1.271	1.932	1.552	1.333

### 3.4 Molecular docking verification

**FIGURE 8:**
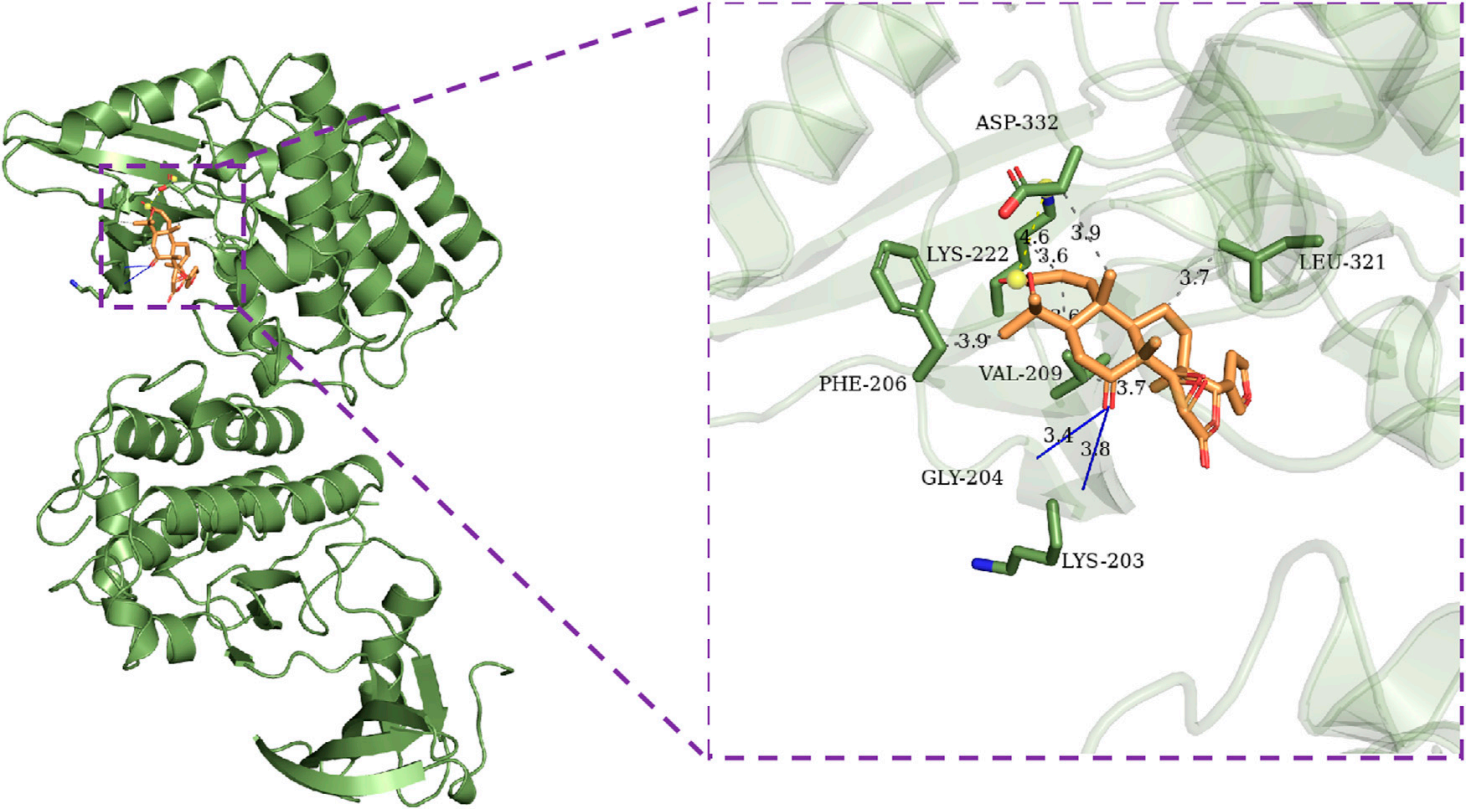
Molecular docking results of Obacunone with SRC (1BYG). Gray dashed lines represent hydrophobic interactions, blue lines represent hydrogen bonding interactions, and yellow dashed lines represent salt bridges.

This section of the study used molecular docking to validate the rationality of EF’s 3D-QSAR pharmacophore model built from ten highly active compounds. The optimal conformation was chosen for each compound, and the RMSD of the conformations was less than 2. As shown in Table 5, the docking scores of the ten active compounds with SRC were all less than -7 kcal.mol^−1^, with the exception of Ethanone. This implies that they play an important role in EF’s anti-cancer process. Supplementary Figure S9 depicts their docking conditions. These compounds all bind to the amino acid residues of SRC *via* hydrophobic effects and hydrogen bonding interactions and have a similar docking mode. More specifically, Obacunone had the highest docking score with SRC, its antihepatoma activity *in vitro* has previously been reported, and its three-dimensional molecular docking state is shown in Figure 8. Obacunone primarily binds to SRC *via* hydrophobic and hydrogen bonding interactions. Obacunone could form hydrophobic interactions with amino acid residues PHE-206, VAL-209, THR-266, and PHE-333, with hydrophobic bond distances of 3.44, 2.61, 3.54, and 3.55 Å, respectively. Meanwhile, obacunone could form hydrogen bonding interactions with GLU-205 and PHE-206 at distances of 2.84 and 3.31 Å, respectively. Obacunone also has one salt bridge interaction with LYS-222. In addition, in order to improve the reliability of molecular docking, some reference drugs were used for docking, such as Ponatinib, Dasatinib, STU (Table 5). Their docking scores were −9.8, −8.7, and −10.9 kcal.mol^−1^, respectively. Some of the screened compounds have affinity values similar to the reference drugs, such as Obacunone, Beta-Sitosterol, Sitosterol, and Berberine. In general, these reference drugs interact with SRC *via* hydrophobic and hydrogen bond interactions. Hydrophobic interactions are formed by amino acid residues ILE-201, VAL-209, TYR-268, and LEU-321, and hydrogen bonds are formed by THR-266 and MET-269. Meanwhile, Ponatinib has some salt bridge interactions (Supplementary Figure S10). Overall, the screened compounds have similar docking modes to the reference drugs, and these docking interactions are consistent with the common features of pharmacophore 01.

**TABLE 5:** The affinity of compounds with SRC(1BYG) (kcal.mol^−1^).

Active compound	Protein(PDBID)	Affinity
24-methyl-31-norlanost-9(11)-enol	SRC(1BYG)	−8.6
Obacunone	SRC(1BYG)	−9.4
beta-Sitosterol	SRC(1BYG)	−9.0
Sitosterol	SRC(1BYG)	−9.3
Fordimine	SRC(1BYG)	−7.8
Rutalinidine	SRC(1BYG)	−8.3
Berberine	SRC(1BYG)	−9.1
Ethanone	SRC(1BYG)	−6.6
Gravacridoneshlirine	SRC(1BYG)	−8.9
Goshuyuamide_I	SRC(1BYG)	−8.7
Ponatinib	SRC(1BYG)	−9.8
Dasatinib	SRC(1BYG)	−8.7
STU	SRC(1BYG)	−10.9

### 3.5 MD simulations

**FIGURE 9:**
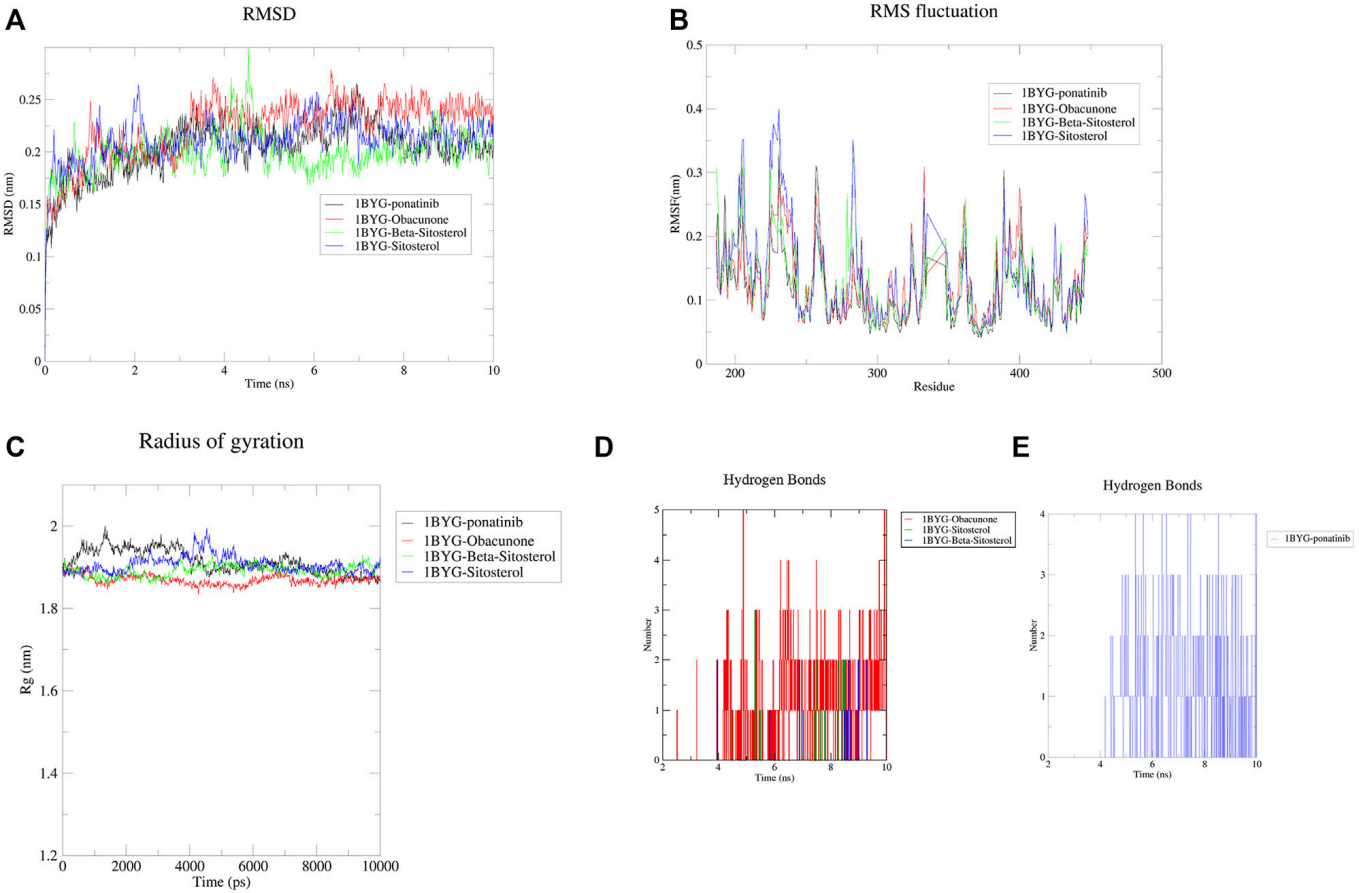
The results of Molecular dynamics simulation. **(A)** RMSD curve of protein-ligand complexes; **(B)** RMSF curve of protein-ligand complexes; **(C)** Radius of gyration of complexes; **(D)** The number of hydrogen bonds formed between the active compounds and 1BYG; **(E)** The number of hydrogen bonds formed between Ponatinib and 1BYG. In **(A–C)**, 1BYG-ponatinib, 1BYG-Obacunone, 1BYG-Beta-sitosterol, and 1BYG-Sitosterol are represented by black, red, green, and blue curves, respectively.

After a 10 ns molecular dynamics simulation, we could further examine how the screened compounds interacted with the protein SRC (1BYG). Obacunone, Beta-sitosterol, and sitosterol were chosen for MD simulation analysis in this work because they had superior docking scores (less than −9 kcal.mol^−1^) than other compounds. Figure 9A shows that throughout the entire procedure, the RMSD of 1BYG Obacunone, 1BYG Beta sitosterol, and 1BYG Sitosterol maintained between .1 and .3, and the fluctuation became stable after 10 ns. It indicates that the 10 ns trajectory conformation of compounds does not reveal substantial structural differences from ponatinib, implying that the ligand and complex structures are stable.

The root mean square fluctuation (RMSF) curve was used to investigate the local changes in protein chain residues. In the 10 ns trajectory files, the fluctuation profiles of the amino acid residues of the complexes (1BYG-Obacunone, 1BYG-Beta-sitosterol, and 1BYG-Sitosterol) were visualized in Figure 9B. RMSF value is lower than 0.4 nm, and a stable fluctuation is generated at about .15, which provides an appropriate basis for subsequent research. 1BYG-obacunone fluctuated surprisingly little in these compounds until about the 300th residue, when it peaked at .3 nm. 1BYG-Beta-sitosterol and 1BYG-Sitosterol have peaks at .32 and .4 nm, respectively with substantial variations in position before residue 300. Their fluctuation trajectories followed the same pattern as 1BYG-Ponatinib.

The radius of gyration (Rg) is a measure of a complex system’s stability in terms of the structural compactness of the molecular dynamics trajectory (Shahbaaz et al., 2019). Rg can also be used to confirm the complex’s stable folding during the simulation. If Rg values are relatively consistent throughout the simulation, the protein structure is considered stable (Ghasemi et al., 2016). A higher Rg value indicates that the protein is more labile, while a lower value indicates that the protein is more stable. In Figure 9C, Rg values of 1BYG-Obacunone were lower and more stable than those of others, this leads in less structural bias and higher stability in the obacunone complexes during simulations.

In addition, we analyzed the number of hydrogen bonds for trajectories lasting 10 ns. Figure 9D shows the hydrogen bonding interaction of obacunone, beta-sitosterol and sitosterol at a distance of 3.5 Å (.35 nm). The maximum number of hydrogen bonds found for obacunone, beta-sitosterol, and sitosterol were 5, 2, and 3, respectively. Over the course of 10 ns, beta-sitosterol, and sitosterol formed fewer hydrogen bonds with 1BYG, indicating poor binding stability. Obacunone formed five hydrogen bonds with 1BYG between 5 and 10 ns, and the number of hydrogen bonds formed was the greatest throughout the simulation process, ensuring better stability. Furthermore, obacunone has a similar density of hydrogen bonding conditions to ponatinib (Figure 9E), which explains its superior binding activity.

## 4 Conclusion

In this study, some potential active compounds such as Obacunone, Beta-sitosterol, Sitosterol, and others were screened using network pharmacology, and SRC was clearly identified as the most promising target of EF in the treatment of liver cancer. The top three signaling pathways are the EGFR signaling pathway, the ErbB signaling pathway, PD-L1 expression, and the PD-1 checkpoint pathway. In addition, the 2-dimensional QSAR pharmacophore model identified 24-methyl-31-norlanost-9(11)-enol, Obacunone, Beta-sitosterol, Sitosterol, Fordimine, Rutalinidine, Berberine, Ethanone, Gravacridoneshlirine, and Goshuyuamide I as highly active compounds. The 3D-QSAR model we created revealed that Pharmacophore 01 had two hydrophobic features and one hydrogen bond acceptor feature. Heatmap and ROC analysis results revealed that pharmacophore 01 possessed high selectivity as well as the ability to distinguish between active and inactive compounds. The molecular docking results confirmed the active compounds’ stable binding to SRC. Furthermore, MD simulations demonstrated the stability of Obacunone, Beta-sitosterol, and Sitosterol in dynamic systems and highlighted Obacunone’s prominent binding capacity. The pharmacophore model proposed in this study provides theoretical support for further screening of effective Chinese medicine compounds against liver cancer and compound structural optimization. Meanwhile, for the optimal compounds, additional pharmacodynamic and pharmacological studies will be conducted to clarify their mechanism of action for the treatment of liver cancer.

## Data Availability

The datasets presented in this study can be found in online repositories. The names of the repository/repositories and accession number(s) can be found in the article/Supplementary Material.

## Abbreviations

BP, biological processes; CC, cell components; C-T-P, compounds-targets-pathways; EF, Evodiae fructus; GFA, genetic function approximation; GO, gene ontology; HCC, hepatocellular carcinoma; ICCA, intrahepatic cholangiocarcinoma; KEGG, kyoto encyclopedia of genes and genomes; MF, molecular functions; MLR, multiple linear regression; PLS, partial least squares; PPI, protein-protein interaction; QSAR, quantitative structure activity relationship; TCM, traditional Chinese medicine.

## References

[B1] Adelusi T. I. Abdul-Hammed M. Idris M. O. Oyedele Q. K. Adedotun I. O. (2021). Molecular dynamics, quantum mechanics and docking studies of some Keap1 inhibitors - an insight into the atomistic mechanisms of their antioxidant potential. Heliyon 7 (6), e07317. 10.1016/j.heliyon.2021.e07317 34195424 PMC8233138

[B2] Ahmed N. Anwar S. Thet Htar T. (2017). Docking based 3D-QSAR study of tricyclic guanidine analogues of batzelladine K as anti-malarial agents. Front. Chem. 5, 36. 10.3389/fchem.2017.00036 28664157 PMC5471292

[B3] Al-Sha'er M. A. Basheer H. A. Taha M. O. (2022). Discovery of new PKN2 inhibitory chemotypes via QSAR-guided selection of docking-based pharmacophores. Mol. Divers. 10.1007/s11030-022-10434-410.1007/s11030-022-10434-4 35507210

[B4] Anwanwan D. Singh S. K. Singh S. Saikam V. Singh R. (2020). Challenges in liver cancer and possible treatment approaches. Biochim. Biophys. Acta Rev. Cancer 1873 (1), 188314. 10.1016/j.bbcan.2019.188314 31682895 PMC6981221

[B5] Bain J. Plater L. Elliott M. Shpiro N. Hastie C. J. McLauchlan H. (2007). The selectivity of protein kinase inhibitors: A further update. Biochem. J. 408 (3), 297–315. 10.1042/bj20070797 17850214 PMC2267365

[B6] Cao W. Chen H. D. Yu Y. W. Li N. Chen W. Q. (2021). Changing profiles of cancer burden worldwide and in China: A secondary analysis of the global cancer statistics 2020. Chin. Med. J. Engl. 134 (7), 783–791. 10.1097/cm9.0000000000001474 33734139 PMC8104205

[B7] Ceppi P. Rapa I. Lo Iacono M. Righi L. Giorcelli J. Pautasso M. (2012). Expression and pharmacological inhibition of thymidylate synthase and Src kinase in nonsmall cell lung cancer. Int. J. Cancer 130 (8), 1777–1786. 10.1002/ijc.26188 21618517

[B8] Chen P. Y. Yuan C. Hong Z. C. Zhang Y. Ke X. G. Yu B. (2021). Revealing the mechanism of "Huai Hua San" in the treatment of ulcerative colitis based on network pharmacology and experimental study. J. Ethnopharmacol. 281, 114321. 10.1016/j.jep.2021.114321 34118340

[B9] Chen Y. Chen X. Ding X. Wang Y. (2019). Afatinib, an EGFR inhibitor, decreases EMT and tumorigenesis of Huh-7 cells by regulating the ERK-VEGF/MMP9 signaling pathway. Mol. Med. Rep. 20 (4), 3317–3325. 10.3892/mmr.2019.10562 31432165 PMC6755195

[B10] Chin C. H. Chen S. H. Wu H. H. Ho C. W. Ko M. T. Lin C. Y. (2014). cytoHubba: identifying hub objects and sub-networks from complex interactome. BMC Syst. Biol. 8 (4), S11. 10.1186/1752-0509-8-s4-s11 25521941 PMC4290687

[B11] Cho J. H. Lee R. H. Jeon Y. J. Shin J. C. Park S. M. Choi N. J. (2015). Role of transcription factor Sp1 in the 4-O-methylhonokiol-mediated apoptotic effect on oral squamous cancer cells and xenograft. Int. J. Biochem. Cell Biol. 64, 287–297. 10.1016/j.biocel.2015.05.007 25982202

[B12] Ditty M. J. Ezhilarasan D. (2021). β-sitosterol induces reactive oxygen species-mediated apoptosis in human hepatocellular carcinoma cell line. Avicenna J. Phytomed 11 (6), 541–550. 10.22038/ajp.2021.17746 34804892 PMC8588954

[B13] Dong L. Shi H. Ji G. Wu D. (2010). Effects of Coptis chinensis and Evodia rutaecarpa water extract on DMH-induced precancerous lesion of colon. Zhongguo Zhong Yao Za Zhi 35 (9), 1185–1188. 10.4268/cjcmm20100923 20707080

[B14] Dumaual C. M. Sandusky G. E. Soo H. W. Werner S. R. Crowell P. L. Randall S. K. (2012). Tissue-specific alterations of PRL-1 and PRL-2 expression in cancer. Am. J. Transl. Res. 4 (1), 83–101. 22347524 PMC3276379

[B15] Dwivedi N. Mishra B. N. Katoch V. M. (2011). 2D-QSAR model development and analysis on variant groups of anti-tuberculosis drugs. Bioinformation 7 (2), 82–90. 10.6026/97320630007082 21938210 PMC3174041

[B16] El-Serag H. B. Marrero J. A. Rudolph L. Reddy K. R. (2008). Diagnosis and treatment of hepatocellular carcinoma. Gastroenterology 134 (6), 1752–1763. 10.1053/j.gastro.2008.02.090 18471552

[B17] Elekofehinti O. O. Iwaloye O. Molehin O. R. Famusiwa C. D. (2021). Identification of lead compounds from large natural product library targeting 3C-like protease of SARS-CoV-2 using E-pharmacophore modelling, QSAR and molecular dynamics simulation. Silico Pharmacol. 9 (1), 49. 10.1007/s40203-021-00109-7 PMC8349134 34395160

[B18] Fathi M. A. A. Abd El-Hafeez A. A. Abdelhamid D. Abbas S. H. Montano M. M. Abdel-Aziz M. (2019). 1, 3, 4-oxadiazole/chalcone hybrids: Design, synthesis, and inhibition of leukemia cell growth and EGFR, Src, IL-6 and STAT3 activities. Bioorg Chem. 84, 150–163. 10.1016/j.bioorg.2018.11.032 30502626 PMC6923798

[B19] Ferreira L. G. Dos Santos R. N. Oliva G. Andricopulo A. D. (2015). Molecular docking and structure-based drug design strategies. Molecules 20 (7), 13384–13421. 10.3390/molecules200713384 26205061 PMC6332083

[B20] Ghasemi F. Zomorodipour A. Karkhane A. A. Khorramizadeh M. R. (2016). *In silico* designing of hyper-glycosylated analogs for the human coagulation factor IX. J. Mol. Graph Model 68, 39–47. 10.1016/j.jmgm.2016.05.011 27356208

[B21] Hajalsiddig T. T. H. Osman A. B. M. Saeed A. E. M. (2020). 2D-QSAR modeling and molecular docking studies on 1H-Pyrazole-1-carbothioamide derivatives as EGFR kinase inhibitors. ACS Omega 5 (30), 18662–18674. 10.1021/acsomega.0c01323 32775868 PMC7407542

[B22] He W. Q. Gao X. Gao L. Ma Y. Sun D. Sun J. (2021). Contrasting trends of primary liver cancer mortality in Chinese mongol and non-mongol. Asian Pac J. Cancer Prev. 22 (9), 2757–2763. 10.31557/apjcp.2021.22.9.2757 34582643 PMC8850897

[B23] Hiscox S. Nicholson R. I. (2008). Src inhibitors in breast cancer therapy. Expert Opin. Ther. Targets 12 (6), 757–767. 10.1517/14728222.12.6.757 18479222

[B24] Hu C. Y. Wu H. T. Su Y. C. Lin C. H. Chang C. J. Wu C. L. (2017). Evodiamine exerts an anti-hepatocellular carcinoma activity through a WWOX-dependent pathway. Molecules 22 (7), 1175. 10.3390/molecules22071175 28708106 PMC6152263

[B25] Huang S. Y. Zou X. (2010). Advances and challenges in protein-ligand docking. Int. J. Mol. Sci. 11 (8), 3016–3034. 10.3390/ijms11083016 21152288 PMC2996748

[B26] Imran S. Taha M. Ismail N. H. Kashif S. M. Rahim F. Jamil W. (2015). Synthesis of novel flavone hydrazones: *In-vitro* evaluation of α-glucosidase inhibition, QSAR analysis and docking studies. Eur. J. Med. Chem. 105, 156–170. 10.1016/j.ejmech.2015.10.017 26491979

[B27] Jiang L. He Y. Luo G. Yang Y. Li G. Zhang Y. (2016). Discovery of potential novel microsomal triglyceride transfer protein inhibitors via virtual screening of pharmacophore modelling and molecular docking. Mol. Simul. 42, 1223–1232. 10.1080/08927022.2016.1149701

[B28] Jin Q. Cheng M. Xia X. Han Y. Zhang J. Cao P. (2021). Down-regulation of MYH10 driven by chromosome 17p13 1 deletion promotes hepatocellular carcinoma metastasis through activation of the EGFR pathway. J. Cell Mol. Med. 25 (24), 11142–11156. 10.1111/jcmm.17036 34738311 PMC8650048

[B29] Kassebaum N. J. Bertozzi-Villa A. Coggeshall M. S. Shackelford K. A. Steiner C. Heuton K. R. (2014). Global, regional, and national levels and causes of maternal mortality during 1990-2013: A systematic analysis for the global burden of disease study 2013. Lancet 384 (9947), 980–1004. 10.1016/s0140-6736(14)60696-6 24797575 PMC4255481

[B30] Li B. Yin X. Cen B. Duan W. Lin G. Wang X. (2022). High value-added application of natural forest product α-pinene: Design, synthesis and 3D-QSAR study of novel α-campholenic aldehyde-based 4-methyl-1, 2, 4-triazole-thioether compounds with significant herbicidal activity. Nat. Prod. Res., 1–6. 10.1080/14786419.2022.211717610.1080/14786419.2022.2117176 36008869

[B31] Li M. Wang C. (2020). Traditional uses, phytochemistry, pharmacology, pharmacokinetics and toxicology of the fruit of tetradium ruticarpum: A review. J. Ethnopharmacol. 263, 113231. 10.1016/j.jep.2020.113231 32758577

[B32] Liao W. Q. Li B. Li L. Zhao J. N. (2014). Study on molecular mechanism of Euodiae Fructus on liver toxicity in MICE. Zhongguo Zhong Yao Za Zhi 39 (24), 1–17. 25898593

[B33] Liu B. Bai C. (2020). Regulatory mechanisms of coicis semen on bionetwork of liver cancer based on network pharmacology. Biomed. Res. Int. 2020, 1. 10.1155/2020/5860704 PMC7700039 33294448

[B34] Luo X. Zheng E. Wei L. Zeng H. Qin H. Zhang X. (2021). The fatty acid receptor CD36 promotes HCC progression through activating Src/PI3K/AKT axis-dependent aerobic glycolysis. Cell Death Dis. 12 (4), 328. 10.1038/s41419-021-03596-w 33771982 PMC7997878

[B35] Ma X. Qiu Y. Zhu L. Zhao Y. Lin Y. Ma D. (2020). NOD1 inhibits proliferation and enhances response to chemotherapy via suppressing SRC-MAPK pathway in hepatocellular carcinoma. J. Mol. Med. Berl. 98 (2), 221–232. 10.1007/s00109-019-01868-9 31872284

[B36] Martin M. W. Newcomb J. Nunes J. J. McGowan D. C. Armistead D. M. Boucher C. (2006). Novel 2-aminopyrimidine carbamates as potent and orally active inhibitors of lck: Synthesis, SAR, and *in vivo* antiinflammatory activity. J. Med. Chem. 49 (16), 4981–4991. 10.1021/jm060435i 16884310

[B37] Meng X. Y. Zhang H. X. Mezei M. Cui M. (2011). Molecular docking: A powerful approach for structure-based drug discovery. Curr. Comput. Aided Drug Des. 7 (2), 146–157. 10.2174/157340911795677602 21534921 PMC3151162

[B38] Meng Y. Zhao Q. An L. Jiao S. Li R. Sang Y. (2021). A TNFR2-hnRNPK Axis promotes primary liver cancer development via activation of YAP signaling in hepatic progenitor cells. Cancer Res. 81 (11), 3036–3050. 10.1158/0008-5472.Can-20-3175 33619115

[B39] Miethe C. Torres L. Zamora M. Price R. S. (2021). Inhibition of PI3K/Akt and ERK signaling decreases visfatin-induced invasion in liver cancer cells. Horm. Mol. Biol. Clin. Investig. 42 (4), 357–366. 10.1515/hmbci-2021-0011 34449178

[B40] Mo C. F. Li J. Yang S. X. Guo H. J. Liu Y. Luo X. Y. (2020). IQGAP1 promotes anoikis resistance and metastasis through Rac1-dependent ROS accumulation and activation of Src/FAK signalling in hepatocellular carcinoma. Br. J. Cancer 123 (7), 1154–1163. 10.1038/s41416-020-0970-z 32632148 PMC7525663

[B41] Muratov E. N. Bajorath J. Sheridan R. P. Tetko I. V. Filimonov D. Poroikov V. (2020). QSAR without borders. Chem. Soc. Rev. 49 (11), 3525–3564. 10.1039/d0cs00098a 32356548 PMC8008490

[B42] Ni Q. Chen Z. Zheng Q. Xie D. Li J. J. Cheng S. (2020). Epithelial V-like antigen 1 promotes hepatocellular carcinoma growth and metastasis via the ERBB-PI3K-AKT pathway. Cancer Sci. 111 (5), 1500–1513. 10.1111/cas.14331 31997489 PMC7226218

[B43] O'Reilly M. Cleasby A. Davies T. G. Hall R. J. Ludlow R. F. Murray C. W. (2019). Crystallographic screening using ultra-low-molecular-weight ligands to guide drug design. Drug Discov. Today 24 (5), 1081–1086. 10.1016/j.drudis.2019.03.009 30878562

[B44] Park S. Y. Park C. Park S. H. Hong S. H. Kim G. Y. Hong S. H. (2017). Induction of apoptosis by ethanol extract of *Evodia rutaecarpa* in HeLa human cervical cancer cells *via* activation of AMP-activated protein kinase. Biosci. Trends 10 (6), 467–476. 10.5582/bst.2016.01170 27890875

[B45] Rampogu S. Son M. Baek A. Park C. Rana R. M. Zeb A. (2018). Targeting natural compounds against HER2 kinase domain as potential anticancer drugs applying pharmacophore based molecular modelling approaches. Comput. Biol. Chem. 74, 327–338. 10.1016/j.compbiolchem.2018.04.002 29702367

[B46] Ren G. Guo J. H. Feng C. L. Ding Y. W. Dong B. Han Y. X. (2022). Berberine inhibits carcinogenesis through antagonizing the ATX-LPA-LPAR2-p38-leptin axis in a mouse hepatoma model. Mol. Ther. Oncolytics 26, 372–386. 10.1016/j.omto.2022.08.001 36090480 PMC9420352

[B47] Ren Z. Schaefer T. S. (2002). ErbB-2 activates Stat3 alpha in a Src- and JAK2-dependent manner. J. Biol. Chem. 277 (41), 38486–38493. 10.1074/jbc.M112438200 11940572

[B48] Roskoski R. Jr. (2015). Src protein-tyrosine kinase structure, mechanism, and small molecule inhibitors. Pharmacol. Res. 94, 9–25. 10.1016/j.phrs.2015.01.003 25662515

[B49] Schenone S. Manetti F. Botta M. (2007). SRC inhibitors and angiogenesis. Curr. Pharm. Des. 13 (21), 2118–2128. 10.2174/138161207781039580 17627544

[B50] Shahbaaz M. Nkaule A. Christoffels A. (2019). Designing novel possible kinase inhibitor derivatives as therapeutics against *Mycobacterium tuberculosis*: An *in silico* study. Sci. Rep. 9 (1), 4405. 10.1038/s41598-019-40621-7 30867456 PMC6416319

[B51] Shan Q. Y. Sang X. N. Hui H. Shou Q. Y. Fu H. Y. Hao M. (2020). Processing and polyherbal formulation of tetradium ruticarpum (A. Juss.) hartley: Phytochemistry, pharmacokinetics, and toxicity. Front. Pharmacol. 11, 133. 10.3389/fphar.2020.00133 32210796 PMC7067890

[B52] Shang N. Wang H. Bank T. Perera A. Joyce C. Kuffel G. (2019). Focal adhesion kinase and β-catenin cooperate to induce hepatocellular carcinoma. Hepatology 70 (5), 1631–1645. 10.1002/hep.30707 31069844 PMC6819211

[B53] Sherman B. T. Hao M. Qiu J. Jiao X. Baseler M. W. Lane H. C. (2022). David: A web server for functional enrichment analysis and functional annotation of gene lists (2021 update). Nucleic Acids Res. 50 (W1), W216–W221. 10.1093/nar/gkac194 35325185 PMC9252805

[B54] Sia D. Villanueva A. Friedman S. L. Llovet J. M. (2017). Liver cancer cell of origin, molecular class, and effects on patient prognosis. Gastroenterology 152 (4), 745–761. 10.1053/j.gastro.2016.11.048 28043904 PMC12160040

[B55] Taha M. O. Al-Sha'er M. A. Khanfar M. A. Al-Nadaf A. H. (2014). Discovery of nanomolar phosphoinositide 3-kinase gamma (PI3Kγ) inhibitors using ligand-based modeling and virtual screening followed by *in vitro* analysis. Eur. J. Med. Chem. 84, 454–465. 10.1016/j.ejmech.2014.07.056 25050878

[B56] Tian Q. Miller E. G. Ahmad H. Tang L. Patil B. S. (2001). Differential inhibition of human cancer cell proliferation by citrus limonoids. Nutr. Cancer 40 (2), 180–184. 10.1207/s15327914nc402_15 11962254

[B57] Tong Z. Li M. Wang W. Mo P. Yu L. Liu K. (2015). Steroid receptor coactivator 1 promotes human hepatocellular carcinoma progression by enhancing wnt/β-catenin signaling. J. Biol. Chem. 290 (30), 18596–18608. 10.1074/jbc.M115.640490 26082485 PMC4513118

[B58] Verma J. Khedkar V. M. Coutinho E. C. (2010). 3D-QSAR in drug design--a review. Curr. Top. Med. Chem. 10 (1), 95–115. 10.2174/156802610790232260 19929826

[B59] Wang H. Wang X. Li X. Zhang Y. Dai Y. Guo C. (2012). QSAR study and the hydrolysis activity prediction of three alkaline lipases from different lipase-producing microorganisms. Lipids Health Dis. 11, 124. 10.1186/1476-511x-11-124 23016923 PMC3567427

[B60] Wang H. Y. Cao Z. X. Li L. L. Jiang P. D. Zhao Y. L. Luo S. D. (2008). Pharmacophore modeling and virtual screening for designing potential PLK1 inhibitors. Bioorg Med. Chem. Lett. 18 (18), 4972–4977. 10.1016/j.bmcl.2008.08.033 18762425

[B61] Xia Z. Tang Z. (2021). Network pharmacology analysis and experimental pharmacology study explore the mechanism of gambogic acid against endometrial cancer. ACS Omega 6 (16), 10944–10952. 10.1021/acsomega.1c00696 34056247 PMC8153951

[B62] Yang F. Shi L. Liang T. Ji L. Zhang G. Shen Y. (2017). Anti-tumor effect of evodiamine by inducing Akt-mediated apoptosis in hepatocellular carcinoma. Biochem. Biophys. Res. Commun. 485 (1), 54–61. 10.1016/j.bbrc.2017.02.017 28189683

[B63] Yang J. Zhang X. Liu L. Yang X. Qian Q. Du B. (2021). c-Src promotes the growth and tumorigenesis of hepatocellular carcinoma via the Hippo signaling pathway. Life Sci. 264, 118711. 10.1016/j.lfs.2020.118711 33186566

[B64] Yoon J. Y. Jeong H. Y. Kim S. H. Kim H. G. Nam G. Kim J. P. (2013). Methanol extract of Evodia lepta displays Syk/Src-targeted anti-inflammatory activity. J. Ethnopharmacol. 148 (3), 999–1007. 10.1016/j.jep.2013.05.030 23747536

[B65] You H. Meng K. Wang Z. Y. (2018). The ER-α36/EGFR signaling loop promotes growth of hepatocellular carcinoma cells. Steroids 134, 78–87. 10.1016/j.steroids.2018.02.007 29481815

[B66] Zhao P. W. Zhang J. W. Liu Y. Liu Y. Liu J. W. Huang J. Z. (2020). SRC-1 and Twist1 are prognostic indicators of liver cancer and are associated with cell viability, invasion, migration and epithelial-mesenchymal transformation of hepatocellular carcinoma cells. Transl. Cancer Res. 9 (2), 603–612. 10.21037/tcr.2019.11.56 35117405 PMC8797421

[B67] Zhao Z. He X. Han W. Chen X. Liu P. Zhao X. (2019). Genus tetradium L.: A comprehensive review on traditional uses, phytochemistry, and pharmacological activities. J. Ethnopharmacol. 231, 337–354. 10.1016/j.jep.2018.11.035 30472402

