# Supplementary material for: Study on the molecular mechanism of anti-liver cancer effect of Evodiae fructus by network pharmacology and QSAR model
Source: Front Chem. 2023 Jan 9;10:1060500. doi: 10.3389/fchem.2022.1060500 (PMC9868320; doi:10.3389/fchem.2022.1060500)
Supplement: Supplementary file 1 [file DataSheet1.docx]

[**Supplementary material**](http://www.rsc.org/suppdata/d0/ra/d0ra06961b/d0ra06961b1.pdf)

content

[1.The tables of Pubchem ID of 27 compounds 2](#_Toc120818854)

[2. The tables of potential therapeutic targets 4](#_Toc120818855)

[3. The tables of GO Enrichment analysis 5](#_Toc120818856)

[4. The tables of degree value of top 10 Potential targets 7](#_Toc120818857)

[5. The tables of key targets by MCC algorithm 8](#_Toc120818858)

[6. ROC analysis 9](#_Toc120818859)

[7. The 27 compounds obtained from TCMSP and TCM-ID database 10](#_Toc120818860)

[8. The figure of venn diagram of coincidence targets 11](#_Toc120818861)

[9. The figure of correlation of GFA on training set compounds 12](#_Toc120818862)

[10. The figure of correlation of MLR on training set compounds 13](#_Toc120818863)

[11. The figure of correlation of PLS on training set compounds 14](#_Toc120818864)

[12. Pharmacophore 02-09 15](#_Toc120818865)

[13. Structural formulae of 35 training set compounds. 16](#_Toc120818866)

[14. Structural formulae of 10 test set compounds. 17](#_Toc120818867)

[15. Structural formulae of 10 test set compounds. 18](#_Toc120818868)

[16. Molecular docking 19](#_Toc120818869)

# 1.The tables of Pubchem ID of 27 compounds

**Table S1** Pubchem ID of 27 compounds

| **Compound ID** | **Compounds Name** | **OB (%)** | **DL** | **Pubchem ID** |
| --- | --- | --- | --- | --- |
| MOL001454 | berberine | 36.86 | 0.78 | 12457 |
| MOL002662 | rutaecarpine | 40.3 | 0.6 | 65752 |
| MOL013352 | Obacunone | 43.29 | 0.77 | 119041 |
| MOL000354 | isorhamnetin | 49.6 | 0.31 | 5281654 |
| MOL000358 | beta-sitosterol | 36.91 | 0.75 | 222284 |
| MOL000359 | sitosterol | 36.91 | 0.75 | 12303645 |
| MOL003942 | Rutaevine | 66.05 | 0.58 | 162989617 |
| MOL003943 | Rutalinidine | 40.89 | 0.22 | 51380995 |
| MOL003947 | 1-methyl-2-[(Z)-pentadec-10-enyl]-4-quinolone | 48.45 | 0.46 | 5319752 |
| MOL003950 | 1-methyl-2-[(Z)-undec-6-enyl]-4-quinolone | 48.48 | 0.27 | 5319810 |
| MOL003957 | 1-methyl-2-pentadecyl-4-quinolone | 44.52 | 0.46 | 5319753 |
| MOL003958 | Evodiamine | 86.02 | 0.64 | 442088 |
| MOL003960 | Ethanone | 30.39 | 0.18 | 624052 |
| MOL003963 | hydroxyevodiamine | 72.11 | 0.71 | 56967381 |
| MOL003964 | 1-methyl-2-undecyl-4-quinolone | 47.59 | 0.27 | 5319811 |
| MOL003972 | 1-methyl-2-nonyl-4-quinolone | 48.42 | 0.2 | 13967189 |
| MOL003974 | Evocarpine | 48.66 | 0.36 | 5317303 |
| MOL003994 | 24-methyl-31-norlanost-9(11)-enol | 38 | 0.75 | 162926950 |
| MOL004004 | 6-OH-Luteolin | 46.93 | 0.28 | 5281642 |
| MOL004014 | Evodiamide | 73.77 | 0.28 | 189454 |
| MOL004017 | Fordimine | 55.11 | 0.26 | 58757248 |
| MOL004018 | Goshuyuamide I | 83.19 | 0.39 | 5317827 |
| MOL004019 | GoshuyuamideII | 69.11 | 0.43 | 5317828 |
| MOL004020 | gossypetin | 35 | 0.31 | 5280647 |
| MOL004021 | Gravacridoneshlirine | 63.73 | 0.54 | 163102888 |
| MOL004025 | N-(2-Methylaminobenzoyl)tryptamine | 56.96 | 0.26 | 5319506 |
| MOL000098 | quercetin | 46.43 | 0.28 | 5280343 |

# 2. The tables of potential therapeutic targets

**Table S2** Potential therapeutic targets

| **Gene Official Symbol** | | | | | | | | |
| --- | --- | --- | --- | --- | --- | --- | --- | --- |
| AURKA | JAK2 | CDK4 | EGFR | MDM2 | NR1H4 | MMP2 | TERT | STAT3 |
| MET | KIT | CASP3 | PYGL | MAPK8 | F2 | ALK | ESR1 | RET |
| PIK3CA | SRC | IDH1 | ERBB2 | MAP2K1 | PIK3R1 | AKT1 | MTOR | SRD5A2 |
| CHEK2 | PTGS2 | AR | MAPK1 | KDR | MMP9 | ABCB1 | FGFR1 | CXCL8 |
| MEN1 | PDGFRA | ROS1 | CCND1 | CYP3A4 | JUN | PTPN11 | BRAF | CASP8 |

# 3. The tables of GO Enrichment analysis

**Table S3** GO Enrichment analysis

| **Category** | **Term** | **Count** | **P-Value** |
| --- | --- | --- | --- |
| BP | GO:0046777~protein autophosphorylation | 13 | 5.34E-15 |
| BP | GO:0018108~peptidyl-tyrosine phosphorylation | 12 | 1.16E-14 |
| BP | GO:0033674~positive regulation of kinase activity | 10 | 6.19E-14 |
| BP | GO:0006468~protein phosphorylation | 16 | 1.58E-13 |
| BP | GO:0007169~transmembrane receptor protein tyrosine kinase signaling pathway | 11 | 3.30E-13 |
| BP | GO:0034614~cellular response to reactive oxygen species | 8 | 1.93E-12 |
| BP | GO:0007275~multicellular organism development | 11 | 4.77E-11 |
| BP | GO:0030335~positive regulation of cell migration | 11 | 2.41E-10 |
| BP | GO:0007165~signal transduction | 18 | 9.15E-10 |
| BP | GO:0043066~negative regulation of apoptotic process | 13 | 1.45E-09 |
| CC | GO:0043235~receptor complex | 11 | 2.64E-11 |
| CC | GO:0032991~macromolecular complex | 13 | 1.88E-08 |
| CC | GO:0005886~plasma membrane | 27 | 1.30E-06 |
| CC | GO:0005654~nucleoplasm | 23 | 4.28E-06 |
| CC | GO:0005829~cytosol | 27 | 6.28E-06 |
| CC | GO:0005634~nucleus | 28 | 7.80E-06 |
| CC | GO:0045121~membrane raft | 7 | 1.53E-05 |
| CC | GO:0005737~cytoplasm | 25 | 1.15E-04 |
| CC | GO:0017053~transcriptional repressor complex | 4 | 3.36E-04 |
| CC | GO:0000791~euchromatin | 4 | 3.36E-04 |
| MF | GO:0004713~protein tyrosine kinase activity | 13 | 2.15E-17 |
| MF | GO:0004714~transmembrane receptor protein tyrosine kinase activity | 13 | 2.97E-17 |
| MF | GO:0005524~ATP binding | 24 | 3.38E-14 |
| MF | GO:0004672~protein kinase activity | 15 | 9.51E-14 |
| MF | GO:0016301~kinase activity | 11 | 8.91E-11 |
| MF | GO:0042802~identical protein binding | 21 | 2.83E-10 |
| MF | GO:0019899~enzyme binding | 11 | 1.73E-08 |
| MF | GO:0005515~protein binding | 45 | 1.73E-08 |
| MF | GO:0019903~protein phosphatase binding | 7 | 7.53E-08 |
| MF | GO:0004674~protein serine/threonine kinase activity | 10 | 2.52E-07 |

# 4. The tables of degree value of top 10 Potential targets

**Table S4** degree value of Top 10 Potential targets

| **Gene official symbol** | **degree** |
| --- | --- |
| SRC | 17 |
| AKT1 | 15 |
| PIK3CA | 14 |
| PIK3R1 | 14 |
| MAPK1 | 13 |
| MAP2K1 | 13 |
| EGFR | 12 |
| JAK2 | 10 |
| MAPK8 | 10 |
| KDR | 10 |

# 5. The tables of key targets by MCC algorithm

**Table S5** key targets by MCC algorithm

| **Rank** | **Gene name** | **Score** |
| --- | --- | --- |
| 1 | CCND1 | 156555420986904.00 |
| 2 | ERBB2 | 156555420940800.00 |
| 3 | MTOR | 156555420900720.00 |
| 4 | SRC | 156554462902566.00 |
| 5 | ESR1 | 156551853519030.00 |
| 6 | STAT3 | 156551853472560.00 |

# 6. ROC analysis

**Table S6** ROC curve analysis of pharmacophore 01-09

| **Validation with Known Actives/Inactives** | | | | | | | | | |
| --- | --- | --- | --- | --- | --- | --- | --- | --- | --- |
| **Pharmacophore** | **Total Actives** | **Total Inactives** | **True Positives** | **True Negatives** | **False Positives** | **False Negatives** | **Sensitivity** | **Specificity** | **ROC** |
| Pharmacophore_1 | 40 | 9 | 39 | 5 | 4 | 1 | 0.97500 | 0.55556 | 0.768 |
| Pharmacophore_2 | 40 | 9 | 36 | 3 | 6 | 4 | 0.97500 | 0.11111 | 0.747 |
| Pharmacophore_3 | 40 | 9 | 34 | 1 | 8 | 6 | 0.85000 | 0.33333 | 0.547 |
| Pharmacophore_4 | 40 | 9 | 33 | 3 | 6 | 7 | 0.82500 | 0.11111 | 0.585 |
| Pharmacophore_5 | 40 | 9 | 40 | 1 | 8 | 0 | 1 | 0.11111 | 0.628 |
| Pharmacophore_6 | 40 | 9 | 35 | 1 | 8 | 0 | 1 | 0.11111 | 0.736 |
| Pharmacophore_7 | 40 | 9 | 40 | 1 | 8 | 0 | 1 | 0.11111 | 0.539 |
| Pharmacophore_8 | 40 | 9 | 40 | 1 | 8 | 0 | 1 | 0.11111 | 0.531 |
| Pharmacophore_9 | 40 | 9 | 40 | 1 | 8 | 0 | 1 | 0.11111 | 0.506 |

# 7. The 27 compounds obtained from TCMSP and TCM-ID database


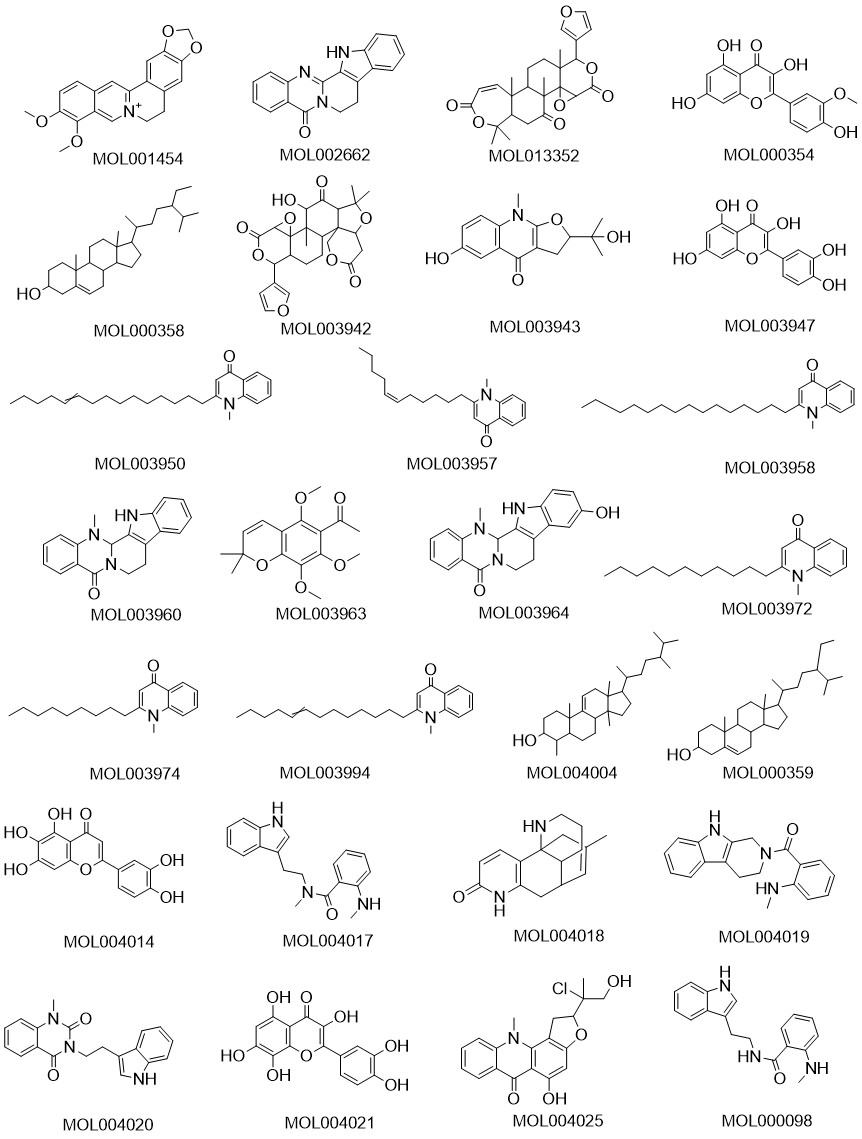


**Figure S1** the 27 compounds obtained from TCMSP and TCM-ID database

# 8. The figure of venn diagram of coincidence targets


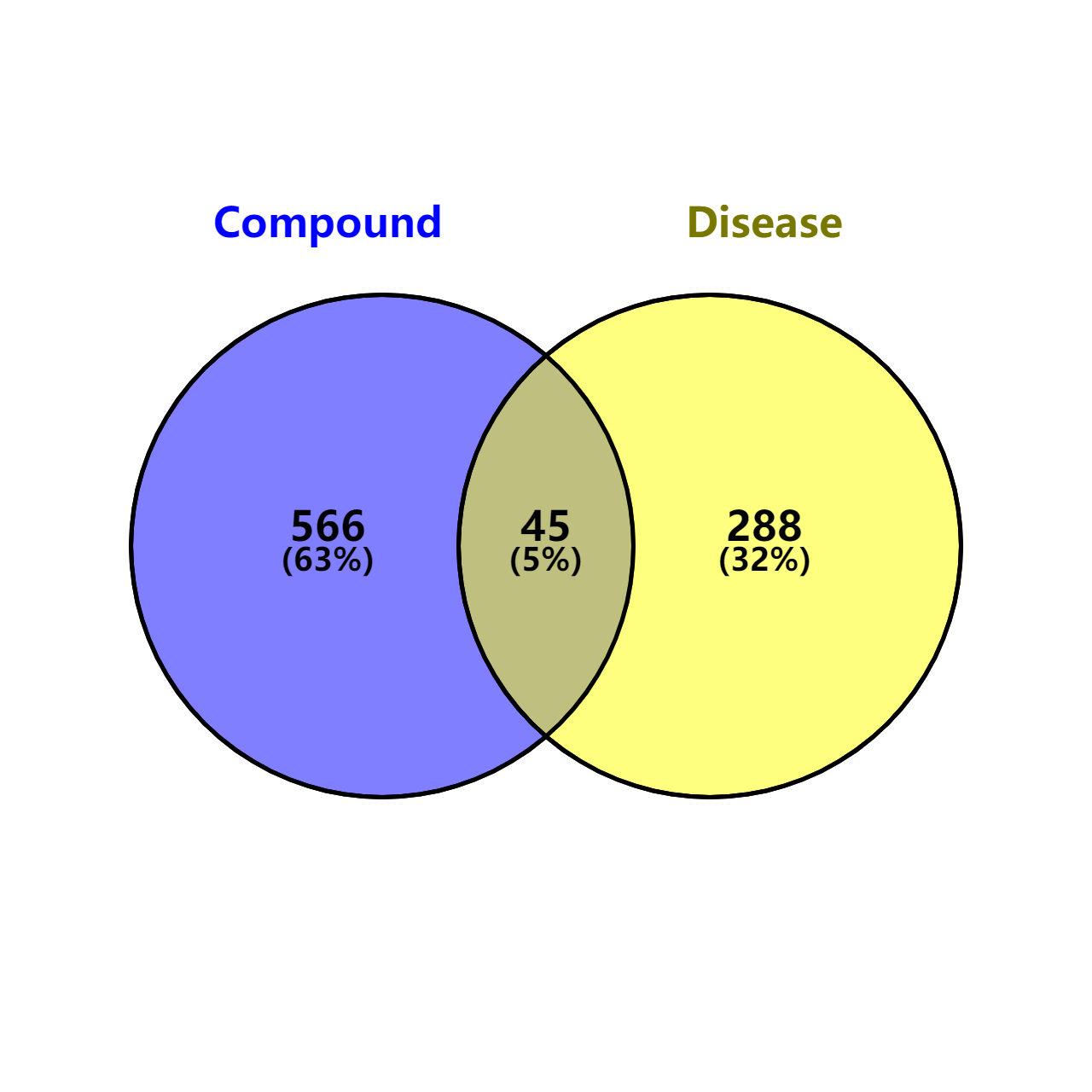


**Figure S2** Venn diagram of coincidence targets

# 9. The figure of correlation of GFA on training set compounds


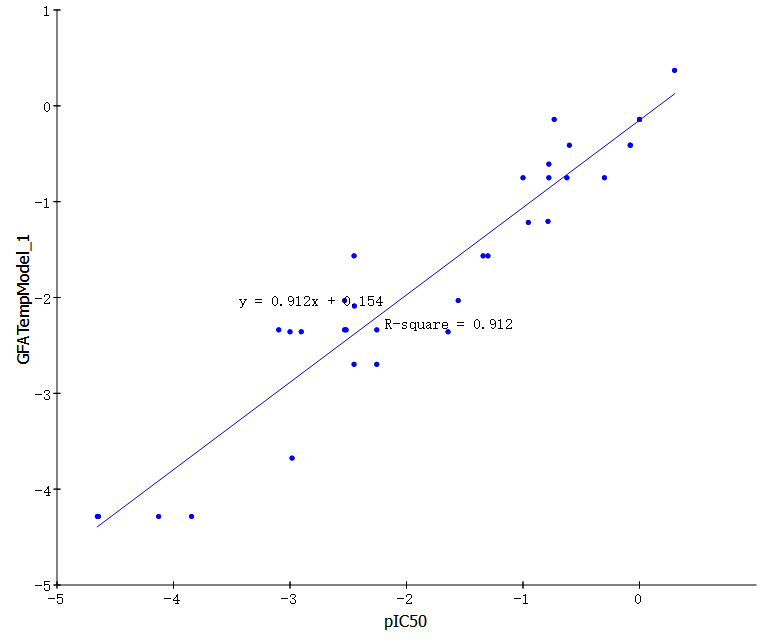


**Figure S3** Correlation between experimental activity and predicted activity (pC50) of GFA on training set compounds.

# 10. The figure of correlation of MLR on training set compounds


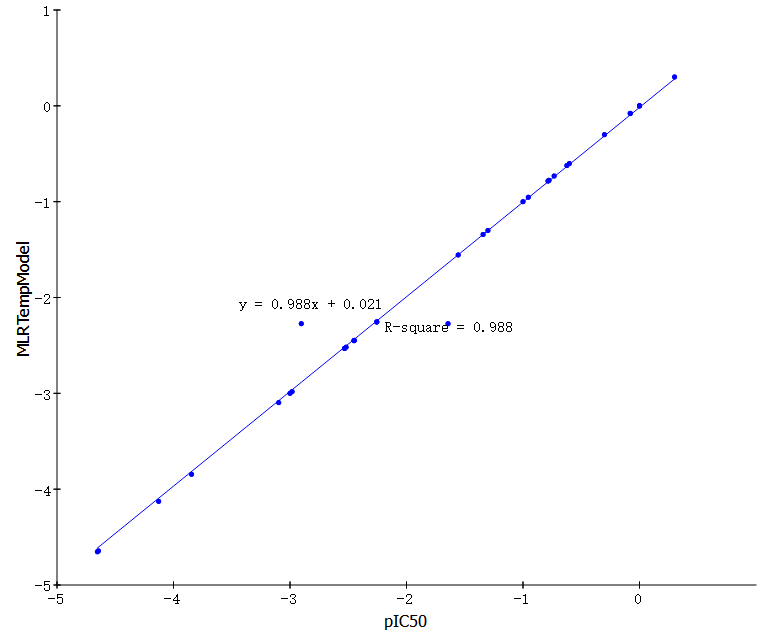


**Figure S4** Correlation between experimental activity and predicted activity (pIC_50_) of MLR on training set compounds.

# 11. The figure of correlation of PLS on training set compounds


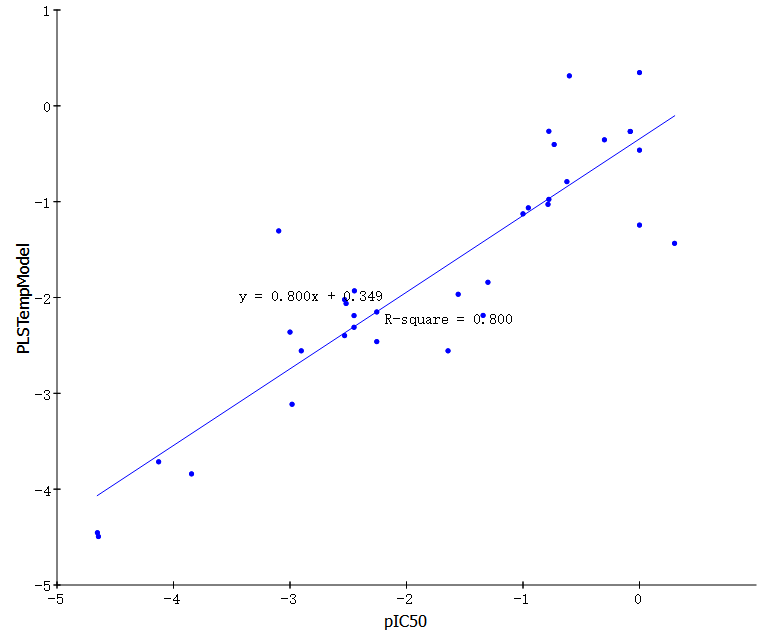


**Figure S5** Correlation between experimental activity and predicted activity (pIC_50_) of PLS on training set compounds.

# 12. Pharmacophore 02-09


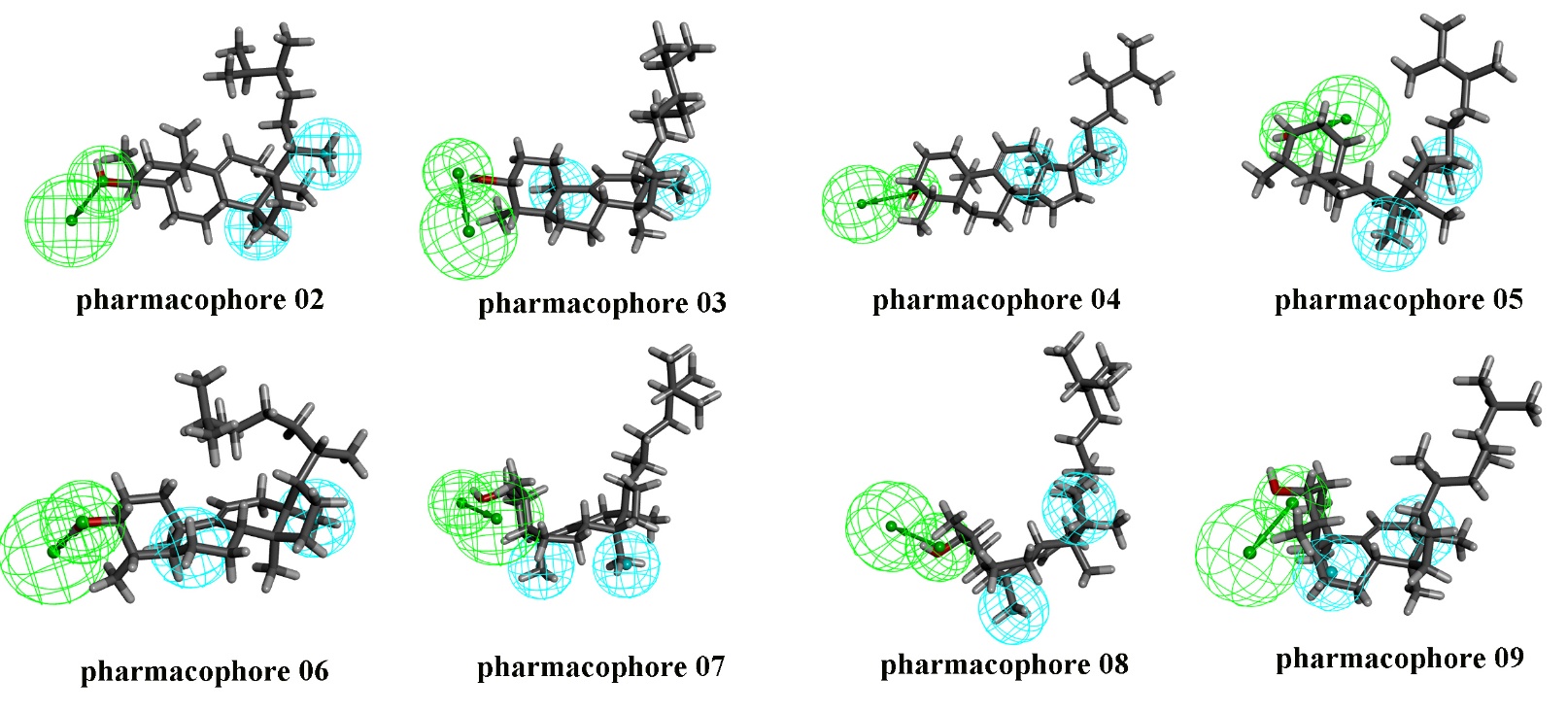


**Figure S6** pharmacophore 02-09

# 13. Structural formulae of 35 training set compounds.

**
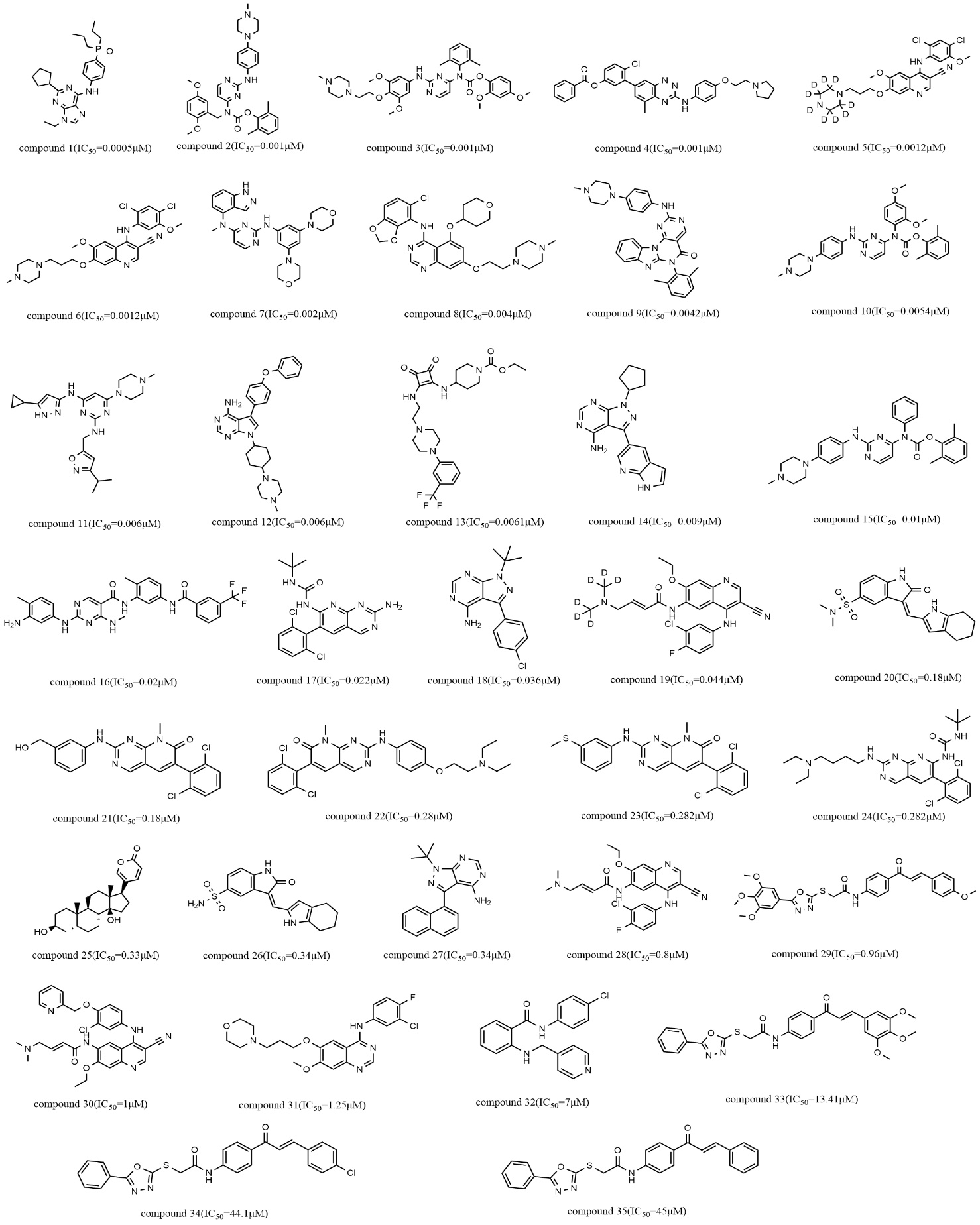
**

**Figure S7** Structural formulae of 35 training set compounds.

# 14. Structural formulae of 10 test set compounds.


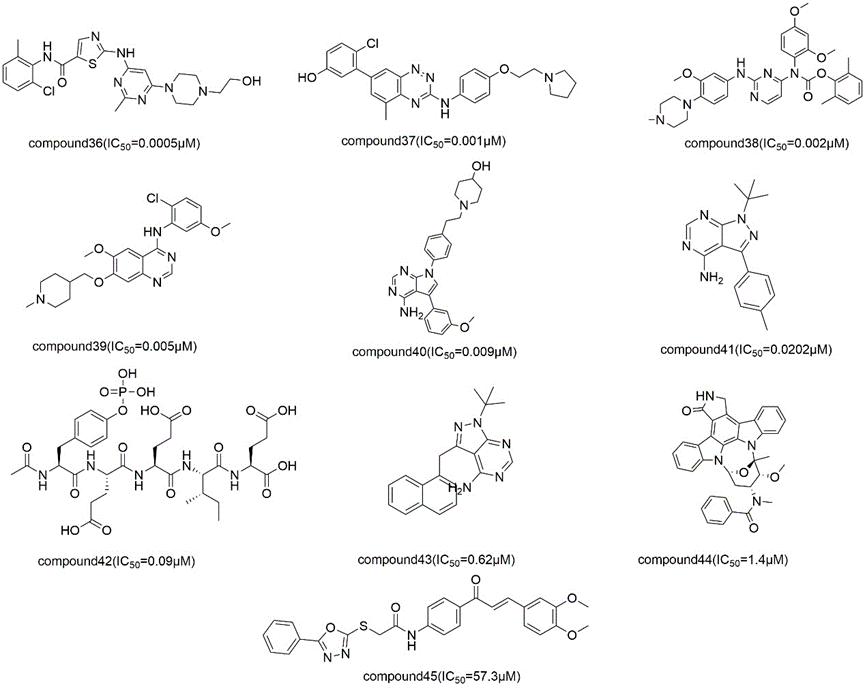


**Figure S8** Structural formulae of 10 test set compounds.

# 15. Structural formulae of 10 test set compounds.


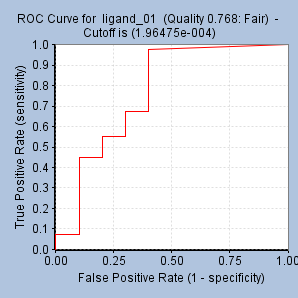


**Figure S9** ROC curve of pharmacophore 01.

# 16. Molecular docking

**
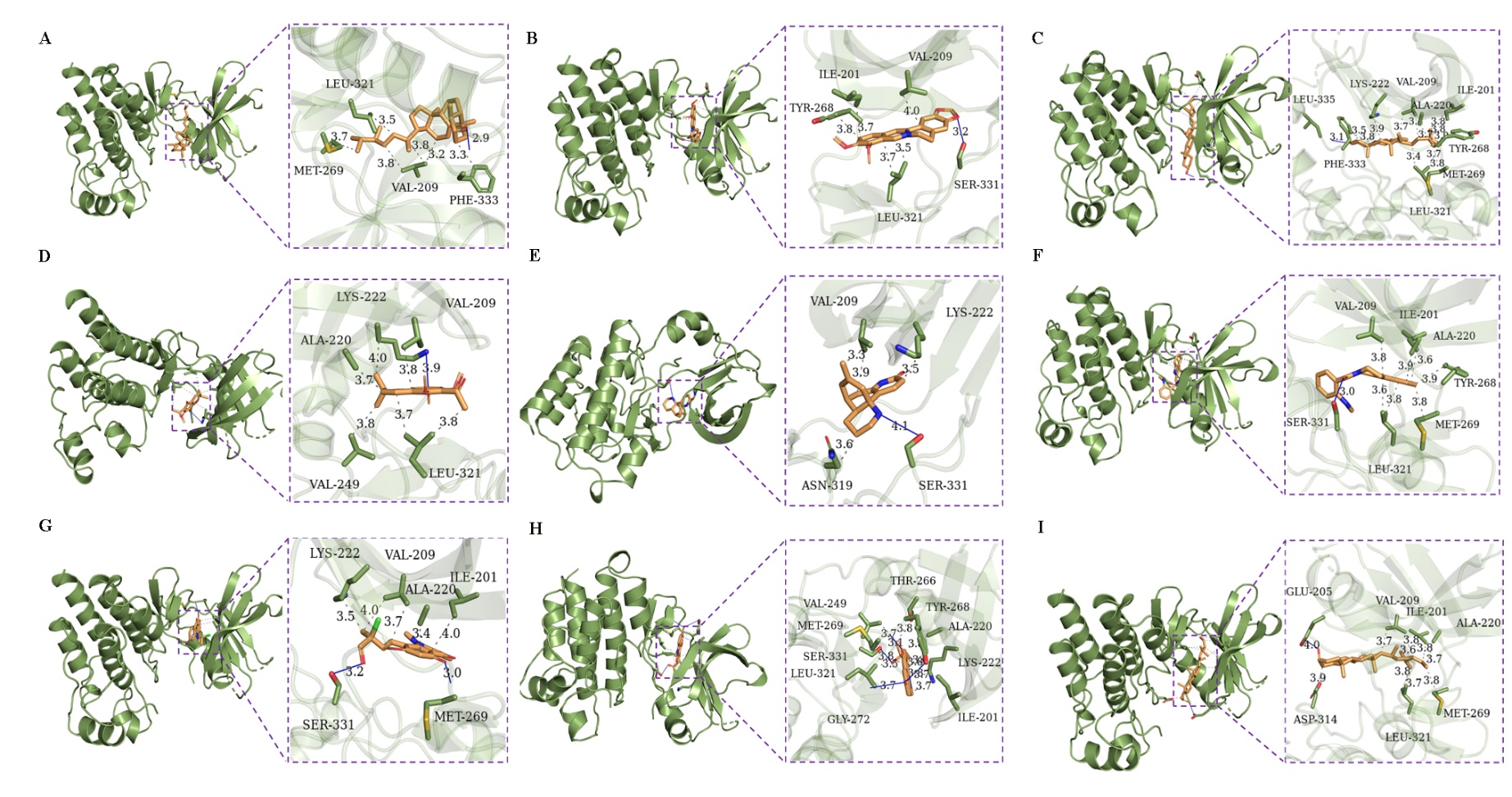
**

**Figure S10** Results of molecular docking, SRC vs A:24-methyl-31-norlanost-9(11)-enol, B: Berberine, C: Beta-sitosterol, D:Ethanone , E:Fordimine, F:Goshuyuamide I, G:Gravacridoneshlirine, H:Rutalinidine, I:Sitosterol.


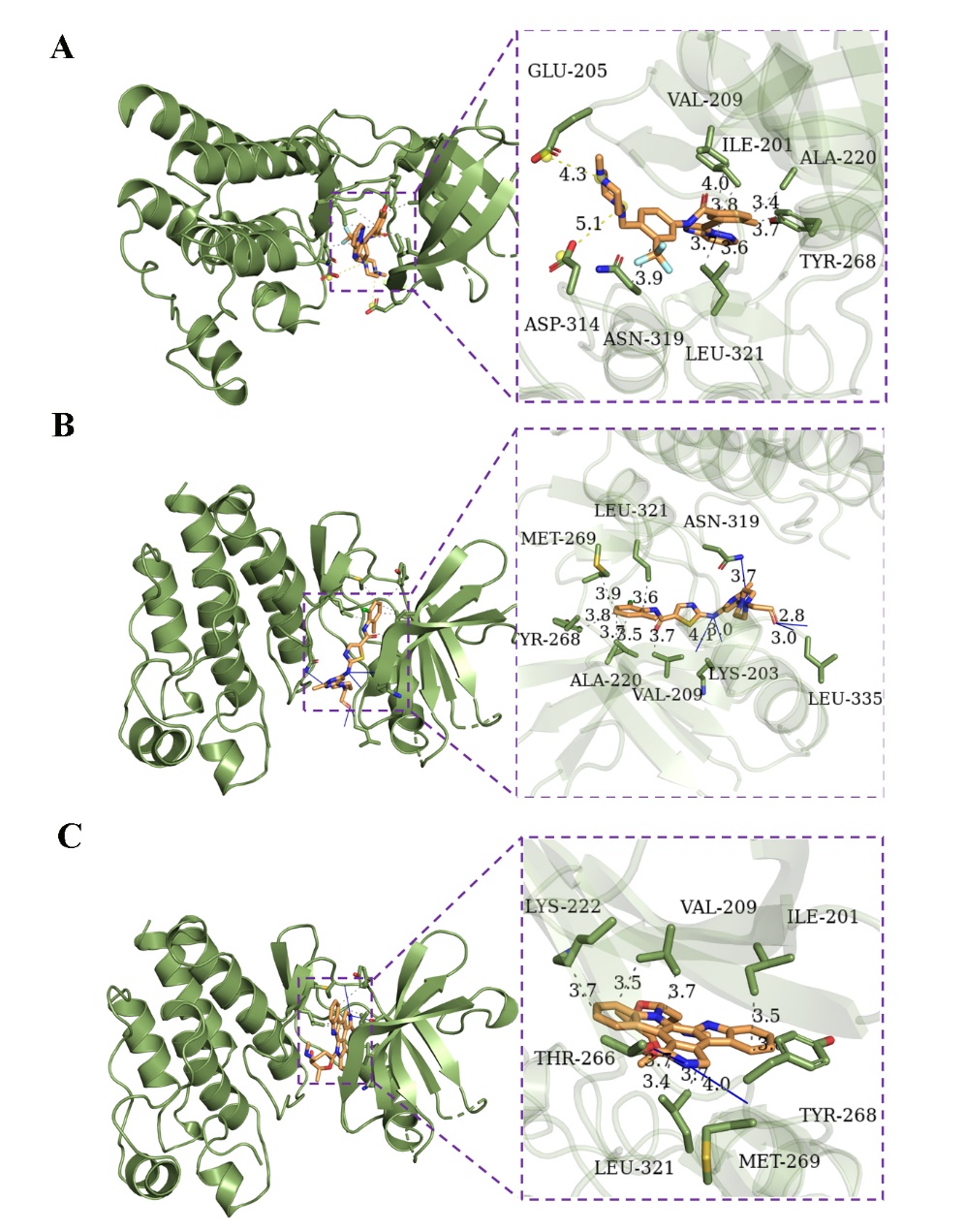


**Figure S11** Results of molecular docking about reference drugs, SRC vs A: Ponatinib, B:Dasatinib, C:STU.
